# Cation- and Anion-Mediated Supramolecular Assembly of Bismuth and Antimony Tris(3-pyridyl) Complexes

**DOI:** 10.1021/acs.inorgchem.1c03004

**Published:** 2021-12-09

**Authors:** Álvaro García-Romero, Jose M. Martín-Álvarez, Daniel Miguel, Dominic S. Wright, Celedonio M. Álvarez, Raúl García-Rodríguez

**Affiliations:** † GIR MIOMeT-IU, Cinquima, Química Inorgánica, Facultad de Ciencias, Universidad de Valladolid, Campus Miguel Delibes, 47011 Valladolid, Spain; ‡ Department of Chemistry, University of Cambridge, Lensfield Road, Cambridge CB2 1EW, U.K.

## Abstract

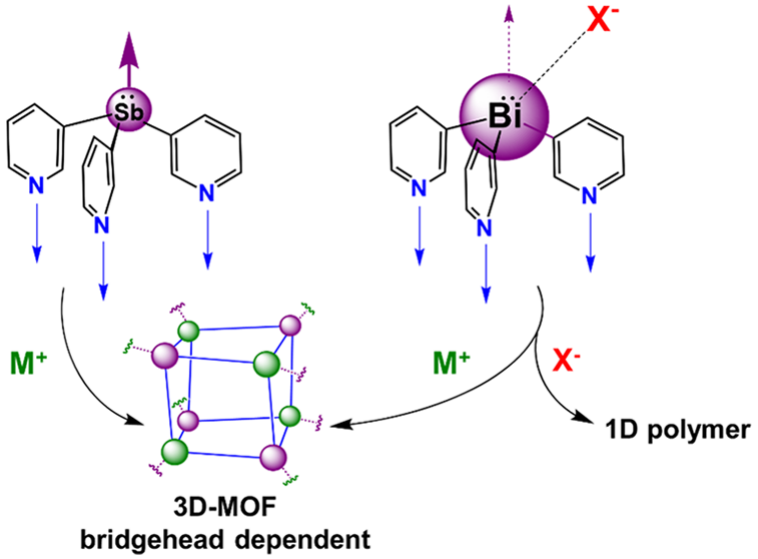

The use of antimony and bismuth in supramolecular chemistry has been largely overlooked in comparison to the lighter elements of Group 15, and the coordination chemistry of the tripodal ligands [Sb(3-py)_3_] and [Bi(3-py)_3_] (L) containing the heaviest p-block element bridgehead atoms has been unexplored. We show that these ligands form a common hybrid metal–organic framework (MOF) structure with Cu(I) and Ag(I) (M) salts of weakly coordinating anions (PF_6_^–^, SbF_6_^–^, and OTf^–^), composed of a cationic substructure of rhombic cage (M)_4_(L)_4_ units linked by Sb/Bi–M bonding. The greater Lewis acidity of Bi compared to Sb can, however, allows anion···Bi interactions to overcome Bi–metal bonding in the case of BF_4_^–^, leading to collapse of the MOF structure (which is also seen where harder metals like Li^+^ are employed). This study therefore provides insight into the way in which the electronic effects of the bridgehead atom in these ligand systems can impact their supramolecular chemistry.

## Abstract 2

The Lewis acidity of the Group 15 bridgehead atoms (E = Sb vs Bi) proves to be a decisive structural directing factor in the coordination of tris(3-pyridyl) ligands E(3-py)_3_, being responsible for promoting or disfavoring E−metal or E···anion interactions.

## Introduction

**Figure 1:**
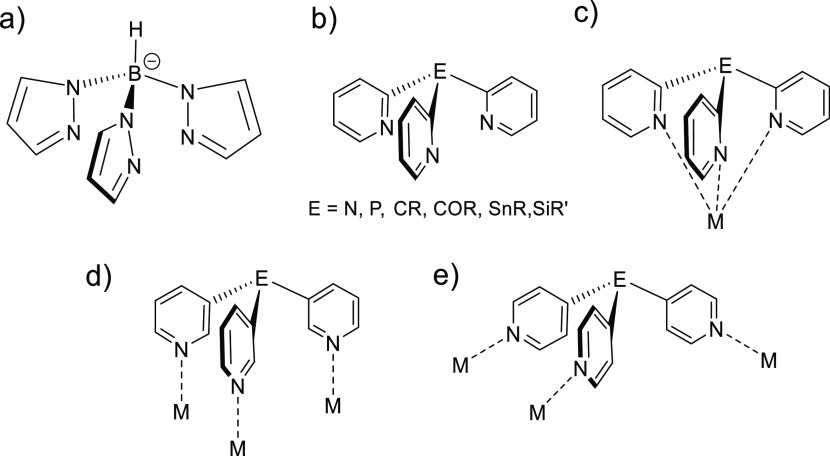
(a) Tris(pyrazolyl) borate ligand framework, (b) nonmetallic “classical” tris(2-pyridyl) ligands, (c) typical N,N,N-coordination mode of E(2-py)_3_ ligands, (d) switch to an intermolecular coordination vector for E(3-py)_3_ ligands, and (e) switching of the coordination vector for E(4-py)_3_ ligands.

Tripodal and facially coordinating ligands have far-reaching applications in modern coordination, organometallic, and bioinorganic chemistry.^1−7^ Tris(pyrazolyl) borates are ideal ligands in this area (Figure 1a), mostly because their electronic and steric characteristics can be easily changed by introducing different substituents within the pyrazolyl ring units, thereby tuning the ligand environment to the particular requirements of the coordinated metal atom.^8^ Recently, there has been a resurgence of interest in closely related tris(2-pyridyl) ligands. In the last 4 decades, studies have focused on tris(2-pyridyl) ligands containing nonmetallic bridgehead atoms, E(2-py)_3_ (E = CR, COR, CH, N, P, P=O, etc.; 2-py = 2-pyridyl; Figure 1b).^9^ However, incorporating heavier and more metallic main-group bridgehead atoms has been shown to provide an important tool for tuning the ligand character, enabling systematic modification of the bite angle, donor/acceptor properties, and reactivity.^10−21^ A case in point is the series of Group 15 tris(2-pyridyl) ligands E(6-Me-2-py)_3_ (6-Me-2-py = 6-methyl-2-pyridyl; E = As, Sb, Bi) for which changing the bridgehead can be used to provide incremental change in the σ-donor character and (thereby) the catalytic activity and behavior.^22^

Most recently, attention has been directed to the effects of moving the donor N atoms from the 2 position to the 3 and 4 positions of the pyridyl ring units.^23−25^ The expected result is that the ligand character switches from the intramolecular N,N,N-chelation mode normally observed for the tris(2-pyridyl) ligands (Figure 1c) to the separate use of the donor N atoms in intermolecular bonding (Figure 1d,e). This modification can therefore be employed as a strategy for the formation of cages and metal–organic frameworks (MOFs). Indeed, the limited studies of the phosphorus ligand P(3-py)_3_ have already indicated how the different coordination vectors of tris(3-pyridyl) ligands can be used in supramolecular chemistry.^26−29^

**Figure 2:**
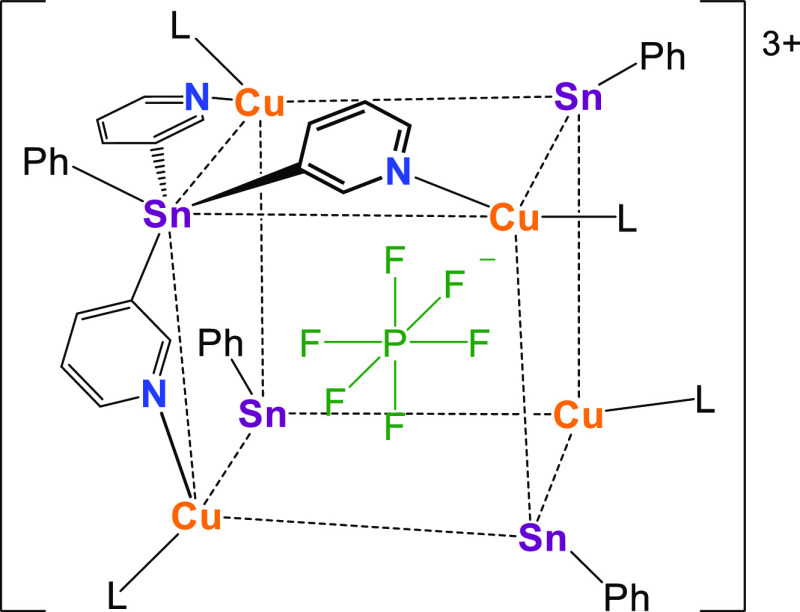
Discrete [{PhSn(3-py)_3_}_4_(Cu·MeCN)_4_(⊂PF_6_)]^3+^ cation found in the structure of the CuPF_6_ complex of PhSn(3-py)_3_ (L = MeCN).

So far, structural studies of the coordination chemistry of tris(3-pyridyl) ligands containing the heavier main-group elements have been limited to the very recently prepared Li[EtAl(3-py)_3_] (whose coordination chemistry was not explored)^30^ and those of Group 14. In accordance with expectations, the MeSi(3-py)_3_^31−33^ and (air-stable) PhSn(3-py)_3_^34^ ligands have been shown to form cage and 1D or 2D network arrangements with metal ions. Particularly relevant to the current study is the cage [{PhSn(3-py)_3_}_4_(Cu·MeCN)_4_(⊂PF_6_)]^3+^, formed by the coordination of CuPF_6_ by PhSn(3-py)_3_ in acetonitrile (MeCN) because of the templating effect of PF_6_^–^.^34^ The cage consists of a roughly cubic Sn_4_Cu_4_ arrangement in which the Sn and Cu atoms form the corners, with a PF_6_^–^ anion being encapsulated in the molecular void (Figure 2).

In the current study, we explore the coordination chemistry of the Group 15 ligands E(3-py)_3_ [E = Sb (**1**), Bi (**2**)] for the first time, with monovalent metal cations (M = Cu^+^, Ag^+^, and Li^+^). Ligands **1** and **2** are observed to adopt either a tridentate N,N,N-bonding mode or a tetradentate N,N,N,E-bonding mode. Strikingly, however, using the N,N,N,E-bonding mode, we are able to combine the ability to form molecular cages with the ability to construct MOF arrangements, resulting in a series of isostructural hybrid MOFs composed of E_4_M_4_ cage units linked by E–M bonding. Unusually, this structure direction appears to be almost completely independent of the anions present, with the same 3D MOF arrangement being formed regardless of the chemical nature and size of the anion. This situation differs from the anion direction commonly found in supramolecular chemistry^35,36^ as well as that seen previously in Group 14 tris(3-pyridyl) metal complexes, in which the chemical and physical characters of the anion normally have profound effects on supramolecular assembly.^34^

## Results and Discussion

### Ligand Synthesis

**Scheme 1:**
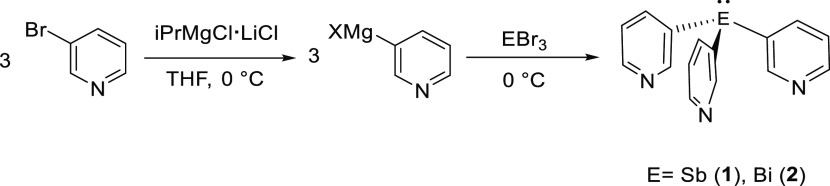
Synthesis of the Heavier Group 15 Ligands **1** and **2**

The first step in our studies was to develop efficient syntheses of the key ligands **1** and **2**. The most obvious route that has been applied to various tris(2-pyridyl) ligands previously, involving reaction of the element trihalides EX_3_ (E = Sb, Bi; X = Cl, Br) with 3-lithiopyridine, failed to produce **1** and **2**, leading to a complex mixture of unidentified products. However, we found that both ligands can be obtained easily via the one-pot reactions of 3-bromopyridine with the turbo-Grignard iPrMgCl·LiCl^37^ at 0 °C in tetrahydrofuran (THF), followed by the addition of EBr_3_ (E = Sb, Bi), with **1** and **2** being isolated in 74 and 80% crystalline yields, respectively (Scheme 1 and the Experimental Section). The reaction time, solvent, and metal halide used were found to be vital to obtaining high yields of **1** and **2**. In particular, the use of ECl_3_ instead of EBr_3_ led to a large reduction in their yields. Previously, the Bi ligand **2** had been prepared using the reaction of BiCl_3_ with the zinc reagent 3-(ZnBr)-pyridine in low yield (20%).^38^ However, this low yield has limited its utility, and therefore no structural studies of its coordination compounds had appeared prior to the current study.

Compounds **1** and **2** are thermally stable and can be stored indefinitely in solid form under a N_2_ atmosphere. They are also remarkably stable toward moisture. No hydrolysis was observed for 1 week after the addition of either ca. 10 equiv of H_2_O in CDCl_3_ or deuterated dimethyl sulfoxide (DMSO-*d*_6_) at room temperature, as monitored by ^1^H NMR spectroscopy.

**Figure 3:**
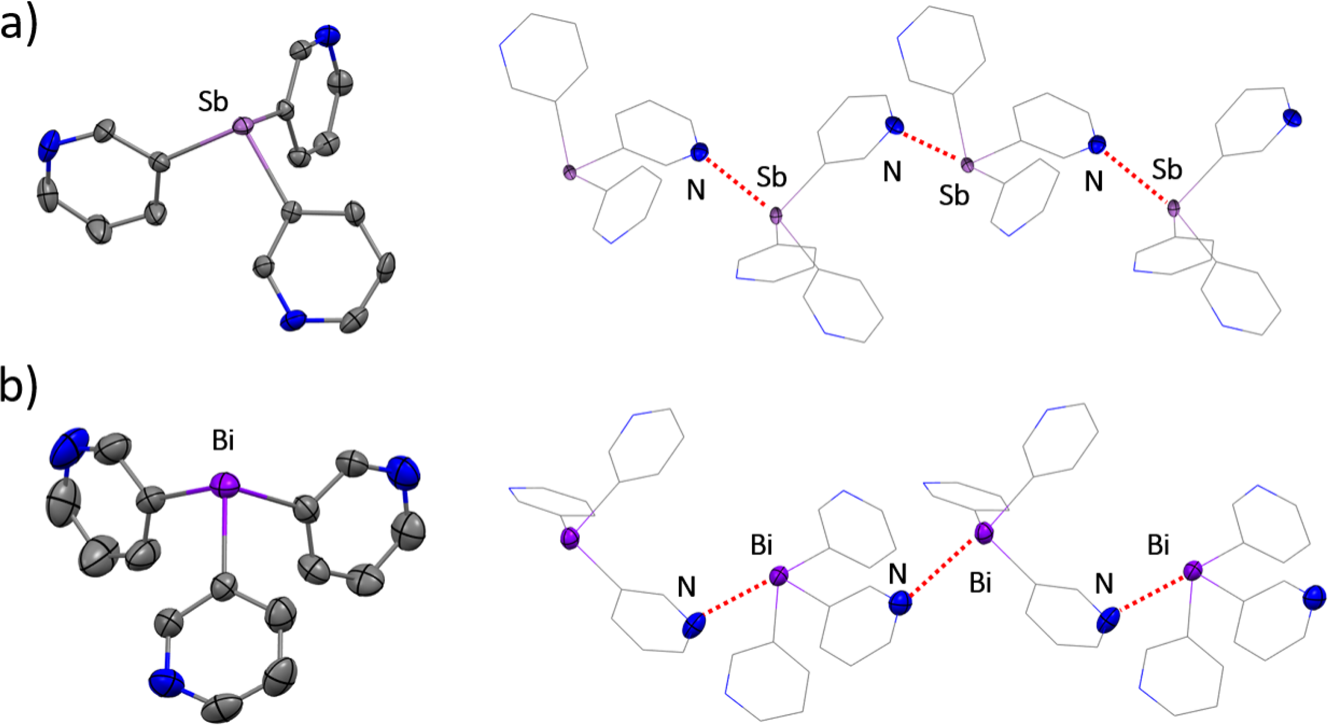
(a, left) Molecular structure of the monomeric unit of **1**. (a, right) Formation of a zigzag-type structure in the crystal lattice through Sb···N interactions between the monomeric units [Sb···N 3.355(4)–3.452(4) Å; cf. 3.61 Å for ∑_VDW_(Sb···N)].^39,40^ (b, left) Molecular structure of the monomeric unit of **2**. (b, right) Formation of a zigzag-type structure in the crystal lattice through Bi···N interactions between the monomeric units [Bi···N 3.374 (4) Å; cf. 3.62 Å for ∑_VDW_(Bi–N)]. Displacement ellipsoids at 50% probability. H atoms and a second independent molecule of **1** present in the asymmetric unit are omitted for clarity. Selected bond lengths (Å) and angles (deg) for **1**: Sb–C_py_ range 2.149(4)–2.160(4); C_py_–Sb–C_py_ range 91.3(1)–98.5(2). Selected bond lengths (Å) and angles (deg) for **2**: Bi–C_py_ range 2.249(4)–2.261(4); C_py_–Bi–C_py_ range 90.5(1)–96.3(1). Color key: C, gray; Sb, light purple; Bi, purple; N, blue.

The single-crystal X-ray structures of both **1** and **2** are shown in Figure 3. As expected, molecules of **1** and **2** show pyramidal Sb(III) and Bi(III) centers. The more acute C–Bi–C angles in **2** [C_py_–Bi–C_py_ range 90.5(1)–96.3(1)°] indicate somewhat more p character in the C–Bi bonds and therefore higher s character in the metal lone pair compared to the Sb derivative [C_py_–Sb–C_py_ range 91.3(1)–98.5(2)°]. Both species form 1D polymeric arrangements as a result of the association of molecules by short intermolecular Sb···N or Bi···N contacts that are well below the sum of their van der Waals radii (Figure 3). However, the Bi···N interactions in **2** [3.374(4) Å] are significantly longer than those recently observed in the structure of Bi(4-py)_3_ [range 3.071(5)–3.306(5) Å], which also forms a polymeric arrangement in the solid state through Bi···N interactions.^25^

### Coordination Chemistry

With ligands **1** and **2** in hand, we moved to explore their coordination chemistry and evaluate the impact of the bridgehead on building supramolecular arrangements. For this purpose, we focused on monovalent cations (Cu^+^, Ag^+^, and Li^+^), which we hoped would facilitate simple comparisons on the basis of the ionic size alone. Studies of Au^+^ proved to be unfruitful because the reactions of **1** and **2** with AuCl(THT) (THT = tetrahydrothiophene) led to complete decomposition. In both cases, 3,3′-bipyridine was observed as a product along with a black precipitate (presumably containing metals; see the Supporting Information, SI).

**Figure 4:**
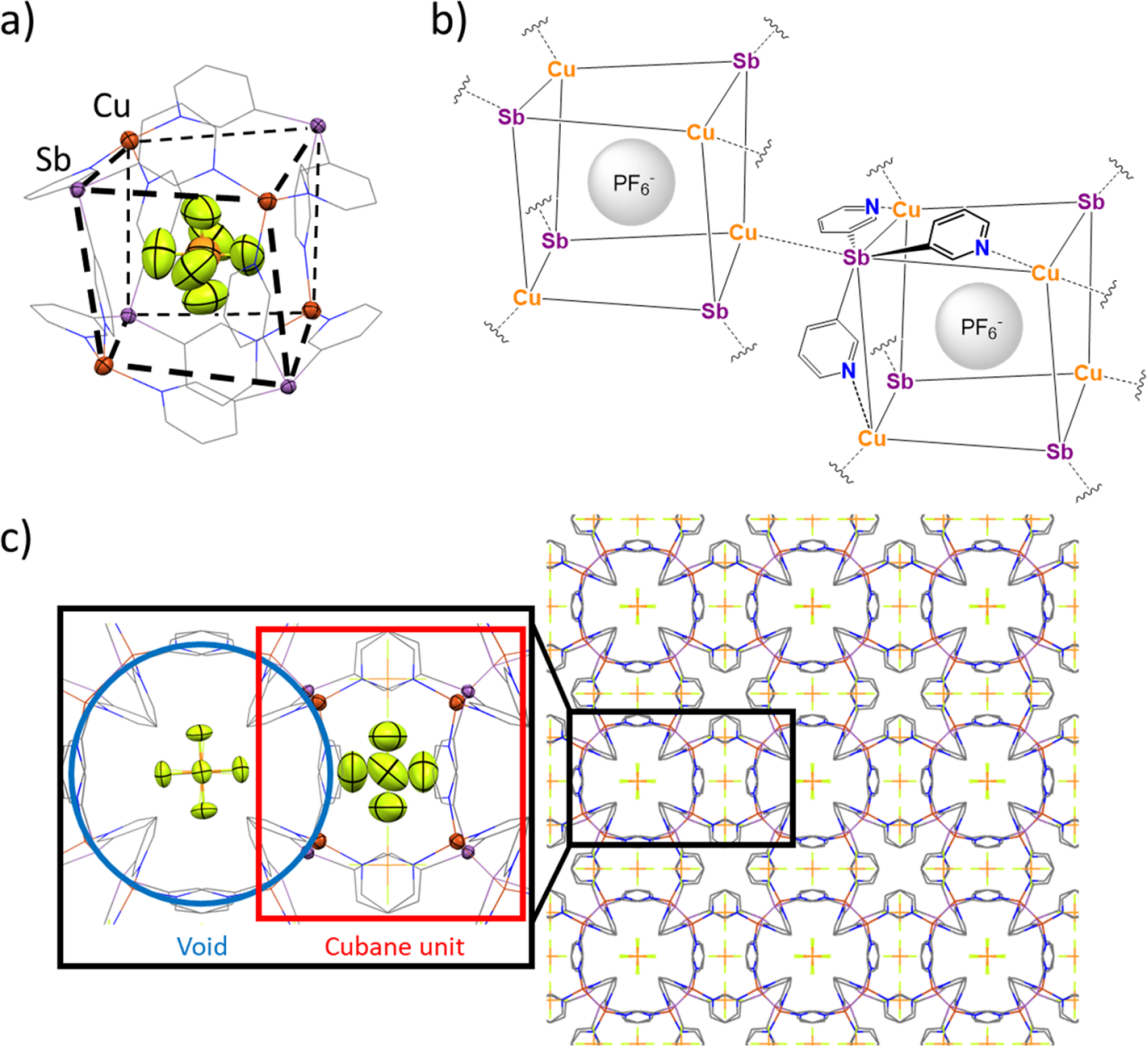
(a) Crystal structure showing the heterocubane monomeric unit [Cu(**1**)(⊂PF_6_)]^3+^ of the 3D MOF [**1**(CuPF_6_)]_*n*_ (**1**·CuPF_6_). This heterocubane unit consists of a cube in which Sb and Cu atoms are placed alternately at the vertices and linked together by a pyridine arm (edge). Displacement ellipsoids at 50% probability. H atoms and *exo*-PF_6_ are omitted for clarity. Selected bond lengths (Å) and angles (deg): Sb–C_py_ 2.132(8); Cu–N 2.051(8); C_py_–Sb–C_py_ 98.7(5); N–Cu–N 110.0(3). (b) Schematic representation of the formation of a polymeric 3D structure in the crystal lattice through Sb–Cu interactions between the monomeric cubane units. (c) Wireframe X-ray structure along the *a*, *b*, or *c* axes of the 3D structure of [**1**(CuPF_6_)]_*n*_. The structure presents a network in which the cubane units (highlighted in red) and voids (highlighted in blue) alternate. One-fourth of the PF_6_^–^ anions are found inside the cubanes, while the other three-fourths are located along the voids. Color key: C, gray; Cu, dark orange; Sb, light purple; N, blue; P, orange; F, yellow.

The slow diffusion of a solution of [Cu(MeCN)_4_]PF_6_ (1 equiv) in MeCN into a dichloromethane (DCM) solution of **1** over 1 week gave yellow blocks of the 3D MOF [**1**(CuPF_6_)]_*n*_ (**1**·CuPF_6_) in 79% yield (Figure 4).The complex crystallizes in the cubic space group *P*4̅3*n*. The X-ray structure shows that four ligands **1** bridge four Cu(I) centers using all three pyridyl arms, forming a cationic [Cu_4_Sb_4_(⊂PF_6_)]^3+^ heterocubane unit in which a PF_6_^–^ ion is encapsulated (Figure 4a). No short contacts were observed between this PF_6_^–^ ion and the surrounding shell of the cage units. These units are isostructural with the discrete cationic cage [{PhSn(3-py)_3_}_4_(Cu·MeCN)_4_(⊂PF_6_)]^3+^ noted previously (Figure 2).^34^ However, because the Sb center of **1** possesses a lone pair [rather than being blocked by a Ph group, as in the case of the PhSn(3-py)_3_ ligand], it is now able to coordinate to a Cu(I) ion of an adjacent cubane unit (Figure 4b). This results in the formation of a 3D MOF in which each cubane is linked to eight surrounding cubane units by Sb–Cu bonds, with the Sb and Cu centers both being four-coordinated (Figure 4c). The Sb–Cu bond lengths [2.596(1) Å] are comparable to the sum of the covalent radii (2.52–2.71 Å for Sb–Cu)^41,42^ and are similar to the donor–acceptor bonds found in stibine–copper complexes, e.g., [Cu(SbPh_3_)_4_][ClO_4_] (2.572–2.577 Å).^43^ The connection of the cubane units produces a cationic sublattice in which voids are formed between the cubane units in which the remaining PF_6_^–^ anions are located.

After 1 week of exposure to air, no loss of crystallinity was observed for **1**·CuPF_6_, and measurement by single-crystal X-ray diffraction showed that no degradation had occurred, demonstrating its stability in the solid state against H_2_O and O_2_. Although **1**·CuPF_6_ is not soluble in common organic solvents (THF, CH_2_Cl_2_, methanol, DMSO, etc.), it is slightly soluble in strongly coordinating pyridine, in which it breaks down into free ligand **1**, as observed by NMR spectroscopy.

### Effect of Other Anions

**Scheme 2:**
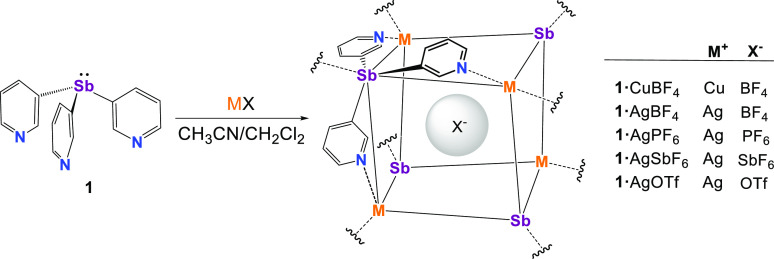
Synthesis of the Copper Complexes **1**·CuX (X = BF_4_^–^, PF_6_^–^) and the Silver Complexes **1**·AgX (X = BF_4_^–^, PF_6_^–^, SbF_6_^–^, OTf^–^)

In order to explore the possible templating effect of the PF_6_^–^ anion on the formation of **1**·CuPF_6_, the coordination chemistry of **1** was investigated with a range of CuX (X = BF_4_^–^) and AgX (X = SbF_6_^–^ PF_6_^–^, BF_4_^–^, CF_3_SO_3_^–^) salts using 1:1 stoichiometries (Scheme 2). The new Cu(I) complex **1**·CuBF_4_ and Ag(I) complexes **1**·AgSbF_6_, **1**·PF_6_, **1**·AgBF_4_, and **1**·AgOTf were all obtained in crystalline form and characterized by X-ray crystallography (see the SI and Scheme 2). All of the compounds are isostructural, showing the same MOF arrangement as that seen in **1**·CuPF_6_. The bulk and crystalline materials were confirmed to be identical for all of the complexes by powder X-ray diffraction (PXRD) and elemental analysis (see the Experimental Section and SI).

**Figure 5:**
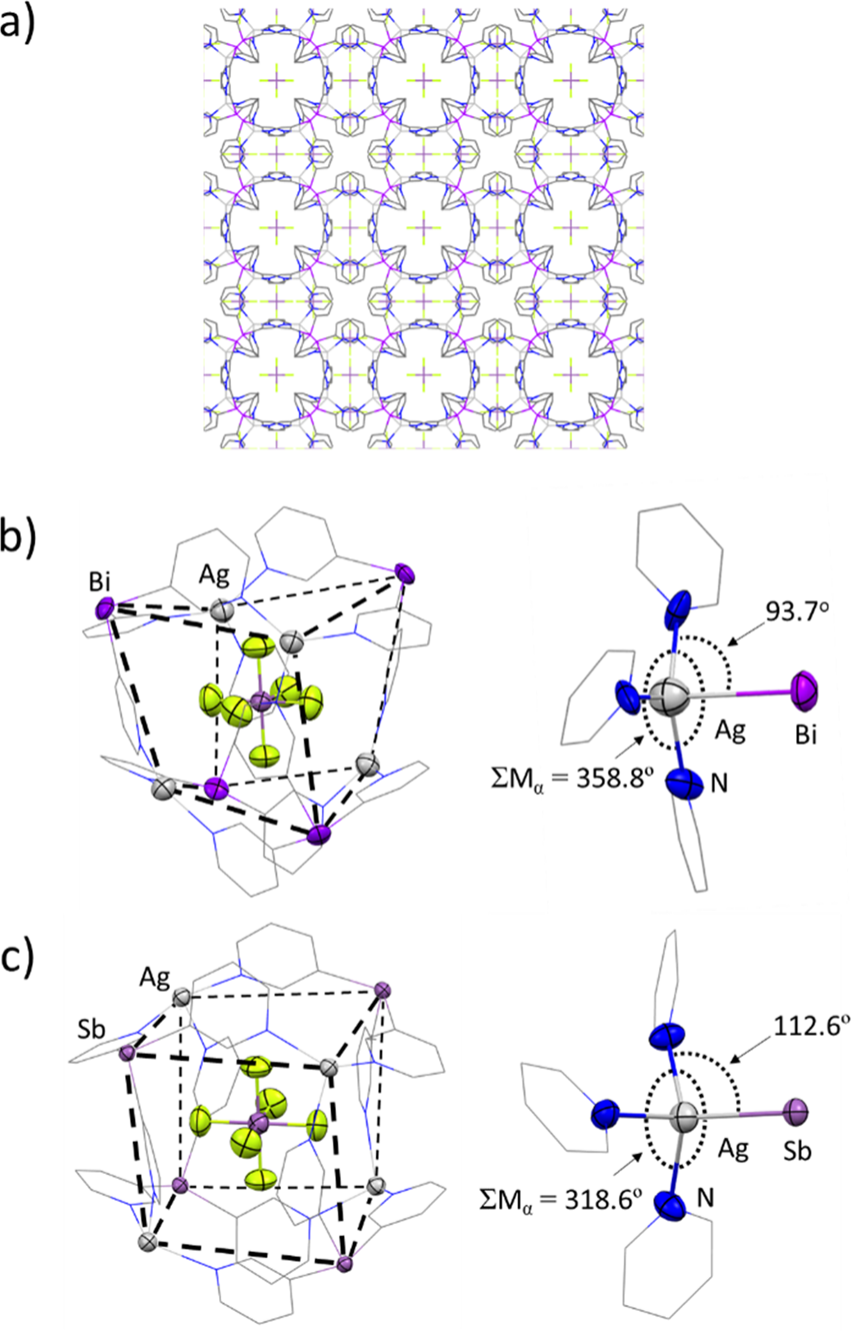
(a) Wireframe view along the *a*, *b*, or *c* axis of the 3D structure of **2**·AgSbF_6_, which consists of a 3D arrangement of [**2**(AgSbF_6_)]_*n*_. The structure presents a network in which the Bi_4_Cu_4_ heterocubane units and the voids are occupied by SbF_6_^–^ anions (1:3 cage/void ratio). Parts b and c show a comparison of the Bi complex **2**·AgSbF_6_ and its Sb analogue **1**·AgSbF_6_. (b, left) X-ray structure fragment showing the cubane unit of the compound **2**·AgSbF_6_. The front and back faces are outlined with dashed lines as a guide to the eye. (b, right) X-ray structure fragment showing the N–Ag–Sb angle. (c, left) X-ray structure fragment showing the cubane unit of **1**·AgSbF_6_. The front and back faces are outlined with blue and red dashed lines, respectively. (c, right) X-ray structure fragment showing the N–Ag–Sb angle. Displacement ellipsoids at 50% probability. H atoms are omitted for clarity. Color key: C, gray; Ag, light gray; Sb, light purple; N, blue; Bi, purple; F, yellow.

Table 1 shows the key cage parameters of the compounds for comparison (including those previously discussed for **1**·CuPF_6_). The Ag–Sb distances linking the cage units in compound **1**·AgX [range 2.654(1)–2.7092(9) Å] are within the sum of the covalent radii (cf. 2.68–2.84 Å for ∑_cov_) and consistent with donor–acceptor interactions, like those found in [Ag(SbPh_3_)_4_][BF_4_] [average 2.724 (Å)].^44^ A common feature of the Sb_4_M_4_ cubane units of the complexes of **1** is their rhombohedral arrangement (formed by six identical, almost planar Sb_2_M_2_ parallelograms of equal Sb···M length; Figure 5c). An interesting observation from these data is that the void volumes of the cage units (range 102.8–138.4 Å^3^) largely correlate with the quantum-chemical-calculated volumes of the encapsulated ions within each series (**1**·CuX and **1**·AgX), i.e., smallest for BF_4_^–^ (*V* = 53.4 Å^3^) and largest for PF_6_^–^ (73.0 Å^3^), OTf^–^ (86.9 Å^3^), and SbF_6_^–^ (88.7 Å^3^)^45^ (see the SI). Indeed, for **1**·AgX, a linear relationship is observed between the cage and anion volumes for all of the anions apart from the prolate OTf^–^ anion (which should, according to this correlation, have an anion volume closer to 105 Å^3^). The observation of the same hybrid MOF structure for all of these compounds using a broad range of weakly coordinating (BF_4_^–^, PF_6_^–^, SbF_6_^–^) and more strongly coordinating (OTf^–^) anions suggests that, although there may be some aspect of anion templating involved, the primary structural direction is the ligand **1** itself and its ability to adopt the N,N,N/Sb-coordination mode.

**Table 1:**
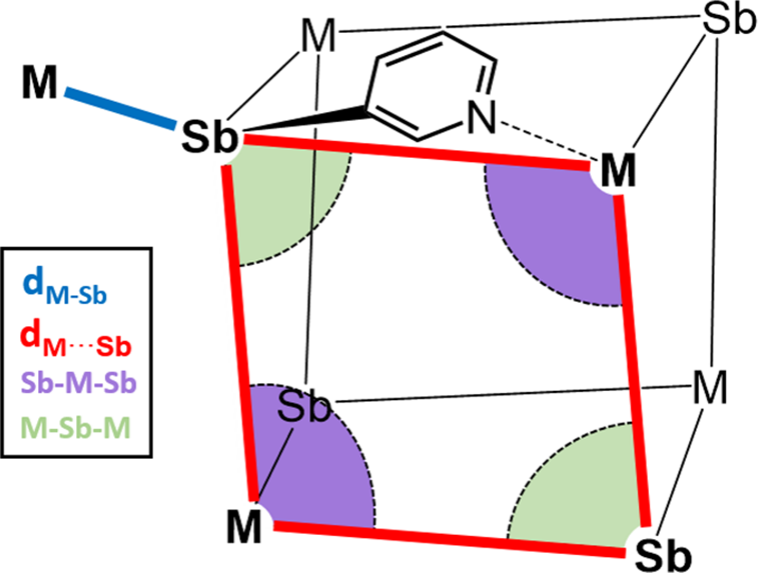
(Top) Representation of the Key Angles and Distances for the Cubane Units in the Sb MOFs and (bottom) Key Cage Parameters for the Metal Cores of the Cu(I) and Ag(I) Complexes of Ligand **1**

compound	M–Sb (Å)	M···Sb (Å)	Sb–M–Sb (deg)	M–Sb–M (deg)	cage void volume (Å^3^)
**1**·CuBF_4_	2.575(1)	6.089(1)	98.29(2)	81.05(2)	125.5
**1**·CuPF_6_	2.596(1)	6.140(1)	98.02(2)	81.36(2)	138.4
**1**·AgBF_4_	2.654(1)	6.183(1)	97.59(2)	81.86(1)	102.8
**1**·AgPF_6_	2.6613(9)	6.2608(9)	96.39(1)	83.23(1)	113.0
**1**·AgSbF_6_	2.6884(9)	6.3183(9)	96.17(1)	83.47(1)	116.1
**1**·AgOTf	2.7092(9)	6.3239(9)	97.24(1)	82.26(1)	123.5

**Scheme 3:**
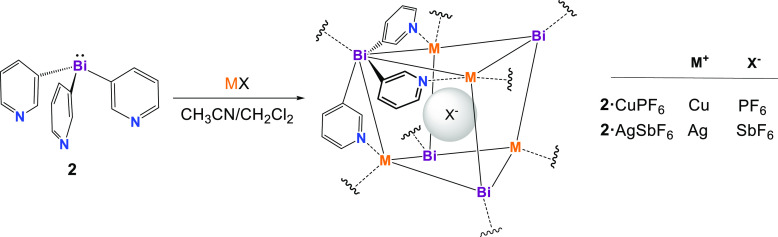
Synthesis of the Bismuthine-Based Copper and Silver Complexes **2**·CuPF_6_ and **2**·AgSbF_6_ Ligand **2** exhibits tetrapodal coordination using a N,N,N/Bi-coordination mode, and each Bi atom is coordinated to a metal, so that each metal is four-coordinate.

In contrast to the Sb(III) bridgehead atom, Bi(III) is less donating and has greater Lewis acidity. We anticipated that this might translate into a stronger interaction with the anions in the case of ligand **2**, promoting N,N,N-tridentate donor behavior by blocking the ability of the Bi lone pair to donate to metal ions. However, the reaction of [Cu(MeCN)_4_]PF_6_ and AgSbF_6_ with **2** resulted in the 3D MOF structures Bi(III)/Cu(I) (**2**·CuPF_6_) and Bi(III)/Ag(I) (**2**·AgSbF_6_), which are closely related to those of ligand **1** (Scheme 3 and Figure 5).

Despite the topological similarity of the MOFs formed with the Sb ligand **1** and the Bi ligand **2**, the cage constituents of **2**·CuPF_6_ and **2**·AgSbF_6_ are markedly more distorted than those of their counterparts **1**·CuPF_6_ and **1**·AgSbF_6_. This can be seen from a comparison of the data in Table 2, which summarizes the key metal core parameters for **2**·CuPF_6_ and **2**·AgSbF_6_, with those in Table 1. Whereas the Sb_4_M_4_ cores of the Cu(I) and Ag(I) complexes of **1** are almost cubic (Figure 5c, left), the Bi_4_M_4_ metal cores of **2**·CuPF_6_ and **2**·AgSbF_6_ are far more distorted, with each pair of opposite (puckered) rhombic faces of the units exhibiting a 90° phase displacement (Figure 5b, left). This greater distortion is largely the result of the rather unusual, almost planar coordination of the pyridyl N atoms to the Cu(I) and Ag(I) centers (Figure 5b, right), in contrast to the pyramidal coordination by the pyridyl N atoms of the metal Cu and Ag in all of the previous compounds of ligand **1** (Figure 5c, right). This is seen from the sums of the N–M–N bond angles (∑M_α_, M = Cu, Ag), which are very close to 360° (∑M_α_ = 356.7° and 358.8° for **2**·CuPF_6_ and **2**·AgSbF_6_, respectively; cf. ∑M_α_ = 330.0° and 318.6° for **1**·CuPF_6_ and **1**·AgSbF_6_, respectively). This planar N coordination and the presence of shorter Cu–N [1.987(6) Å] and Ag–N [2.21(2) Å] bonds in **2**·CuPF_6_ and **2**·AgSbF_6_, respectively, compared to those in their counterparts **1**·CuPF_6_ and **1**·AgSbF_6_ [2.051(8) and 2.305(8) Å, respectively], are symptomatic of the weakness of the Bi–Cu and Bi–Ag bonds compared to the Sb–Cu and Sb–Ag bonds. This is an expected consequence of the relativistic stabilization of the 6s^2^ lone pair on Bi (compared to the greater degree of s–p mixing of the lone pair on the Sb analogue), making the Bi ligand **2** a weaker σ donor [as confirmed by density functional theory (DFT) calculations, discussed later]. A further potential reason for distortion of the cage units of **2**·CuPF_6_ and **2**·AgSbF_6_ is the significantly smaller ligand bite angle of the Bi ligands **2** compared to **1** [e.g., C–Bi–C 94.3(3)–93.9(8)° in **2**·CuPF_6_ and **2**·AgSbF_6_; cf. 98.7(3)–100.4(3)° in **1**·CuPF_6_ and **1**·AgSbF_6_]. It can be noted that there are few examples of Bi–Cu- and Bi–Ag-bonded compounds known in which Bi behaves as an L-type σ donor. In fact, closed-shell Bi interactions with late transition metals have been almost completely unexplored, with only a few examples using the PBiP pincer ligands (P = phenylenephosphino groups) reported by Gabbaï and Limberg, which place the Bi center in close proximity to the metal.^46,47^ Compared to these systems, **2**·CuPF_6_ possesses a similar Bi–Cu bond length but is an example of an unsupported Bi–Cu bond. The Bi–Cu distance of 3.041(2) Å is at the upper limit of the Bi–Cu bond distances reported by Gabbaï and Limberg (2.790–3.095 Å)^48−50^ and lies between the sum of the van der Waals and covalent radii (cf. 3.47 Å for ∑_VDW_ and 2.63–2.80 Å for ∑_cov_). Similarly, the Bi–Ag distance in **2**·AgSbF_6_ [3.178(2) Å] lies between the sum of the van der Waals and covalent radii (cf. 3.79 Å for ∑_VDW_ and 2.63–2.80 Å for ∑_cov_) and is comparable to that reported by Gabbaï and Limberg using their pincer ligands (2.946–3.173 Å).^48−50^

**Table 2:** Key Cage Parameters for the Metal Cores of **2**·CuPF_6_ and **2**·AgSbF_6_^a^

compound	M–Bi (Å)	M···Bi (Å)	Bi–M–Bi (deg)	M–Bi–M (deg)	cage void volume (Å^3^)
**2**·CuPF_6_	3.041(2)	6.065(2)	107.10(2)	69.60(2)	116.6
**2**·AgSbF_6_	3.178(2)	6.260(3)	108.73(4)	67.14(3)	109.9

a The definitions of key angles and distances are analogous to those of the Sb MOFs (Table 1).

### Computational Analysis

The interaction between the Ag and Bi atoms in **2**·AgSbF_6_ was studied using DFT. It should be kept in mind that the previously discussed extended 3D MOF structures are not amenable to simple calculational analysis. To approach this study with low computational cost, we employed a simple model by taking a fragment of the extended X-ray structure consisting of a [Ag(py)_3_]^+^ moiety attached to a Bi(3-py)_3_ ligand (see the SI). When free relaxation of the structure was allowed using the APFD functional, the minimum energy structure exhibited a Ag–Bi distance of 2.655 Å [very similar to that obtained with other functionals (see the SI) but shorter than that observed experimentally, 3.178(2) Å]. The optimized structure shows pyramidalization of the Ag center (∑M_α_ = 341.4°), in contrast to the planar geometry observed in the X-ray structure (∑M_α_ = 358.8°). This suggests that there are other factors affecting the interaction between the Ag and Bi atoms. The structure was then optimized with the same (planar) geometry as that found in the X-ray structure of **2**·AgSbF_6_ at the Ag center. The Ag–Bi bond length was increased to 3.124 Å, which is in very good agreement with the experimental distance. The energy difference between the two models in which Ag is pyramidal or planar is very small (0.6 kcal/mol). Therefore, distortion of the geometry to a planar geometry at Ag observed in the solid-state structure is probably the result of packing effects alone. The freely optimized geometry exhibits a Bi–Ag bond energy of 27.0 kcal/mol, and the X-ray structure model with the planar geometry at Ag shows a Bi–Ag bond energy of 14.1 kcal/mol, in accordance with the larger Bi–Ag distance [both were calculated using an energy decomposition analysis (EDA) performed with the *AOMIX* program].^51^

The DFT calculations also show that the bismuthine ligand **2** acts as a σ donor in the Bi–Ag bonding. In order to identify which orbitals are involved, a natural bonding orbital (NBO) analysis was carried out on the X-ray structure model. The study indicated one main interaction between the filled lone pair on Bi [6s (80%)–6p_*z*_ (20%)] and the empty atomic orbital of Ag [5s (98%)]. No donation from Ag to Bi was identified, indicating that bismuth does not act as an acceptor. Similar calculations using a model system for the X-ray geometry of **1**·AgSbF_6_ show that the Sb–Ag interaction is much stronger (35.2 vs 14.1 kcal/mol for Bi–Ag) and results from one main interaction between the filled lone pair on Sb [5s (66%)–5p_*z*_ (34%)] and the empty atomic orbital of Ag [5s (100%)]. Thus, the weakness of the Bi–Ag bond provides an explanation for the observed and unusual planarity of the Ag center in **2**·AgSbF_6_ and the pyramidalization of the Ag center in **1**·AgSbF_6_. Similar DFT studies show that the same tendency is maintained in the Cu structures, with stronger Sb–Cu interaction (34.2 kcal/mol, 69% 5s + 31% 5p → 100% 5s) relative to the Bi–Cu interaction (15.0 kcal/mol, 79% 6s + 21% 6p → 98% 5s); thus, similarly to the Ag complexes, the planarity Cu^+^ in **2**·CuPF_6_ can be related to the weak Bi–Cu bond. The DFT studies reflect the reluctance of Bi to engage in s–p mixing, which, along with the weaker nature of the M–Bi interaction, provides an explanation for the differences in the Bi-based MOFs compared to the Sb ones and suggests that the Sb ligand **1** should have a larger structure-directing influence than the Bi ligand **2**. It is obvious that the different bridgeheads may lead to very different geometric profiles for the E(3-py)_3_ ligands, but changing the bridgehead can also have large electronic implications. This feature has also recently been highlighted in a study of the formation of 2D or 3D networks using Group 15 tris(4-pyridyl) ligands.^25^

Preliminary studies indicate that the bridgehead–metal interaction also has a large impact on the luminescence properties of the 3D MOFs. For instance, when excited with UV light (λ_ex_ = 375 nm) at room temperature, the Bi(III)/Cu(I) MOF **2**·CuPF_6_ exhibits bright-green luminescence (λ_max,em_ = 530 nm), while its Sb analogue **1**·CuPF_6_ shows red-shifted luminescence in the orange region (λ_max,em_ = 570 nm; see the SI). In both cases, the solid-state photoluminescence emission spectra appear as broad featureless bands. Interestingly, the emission spectra of **1**·CuPF_6_ and **1**·CuBF_4_ are almost identical (see the SI), strongly suggesting that the Group 15–Cu bonding has a major influence on the luminescence and that the luminescence properties can be tuned by these interactions in the solid-state structures of the MOFs.

### Effect of Bi–Anion Interactions

**Figure 6:**
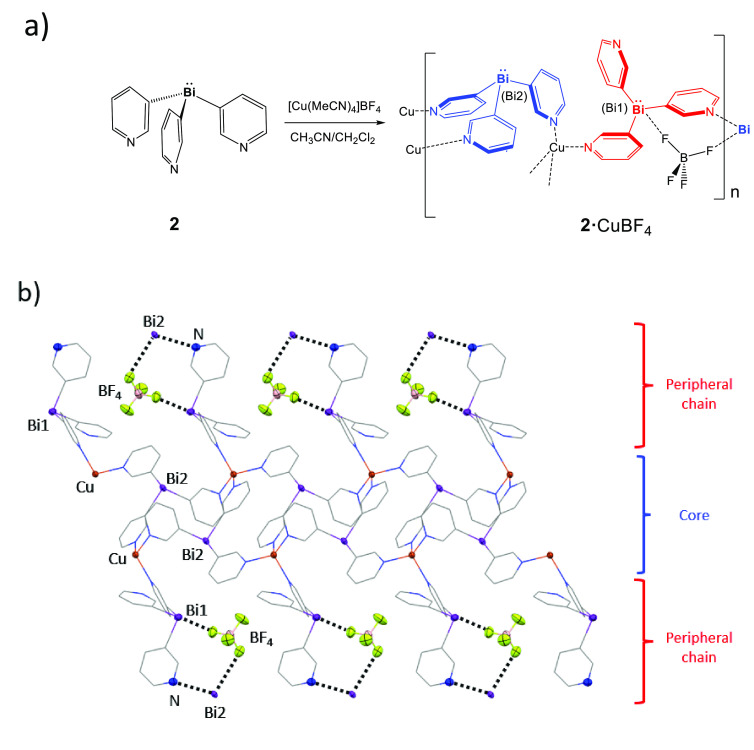
(a) Synthesis of **2**·CuBF_4_. (b) X-ray structure of the extended 1D coordination polymer of **2**·CuBF_4_. H atoms and some of the BF_4_^–^ anions in the lattice (those located in the core) are omitted for clarity. Selected bond lengths (Å) and angles (deg): Bi–C_py_ 2.26(1)–2.29(1); Cu–N 2.03(1)–2.07(1); C_py_–Bi–C_py_ 91.1(5)–95.3(5); N–Cu–N 105.7(4)–114.9(4). Color key: C, gray; Cu, orange; N, blue; Bi, purple; B, pink; F, yellow.

The structural effect of changing the bridgehead can be observed in the reaction of **2** with CuBF_4_. The 1:1 reaction leads to the formation of a 1D coordination polymer [Cu(**2**)_2_][BF_4_] (**2**·CuBF_4_) as yellow needle crystals (rather than cubic crystals as in the previous cases for the 3D MOFs). Although the complex contains a 1:2 ratio of ligand **2** to CuBF_4_, its formation is observed irrespective of the stoichiometry used (1:1 or 1:2), but the use of the required 1:2 stoichiometry leads to higher yields. The X-ray study reveals a complicated structure consisting of a 1D coordination polymer formed by three parallel chains in which no Bi coordination to the Cu(I) center is observed (Figure 6). In the central chain, bridging ligands **2** (Bi2 atom in Figure 6) link Cu(I) units into a polymeric arrangement using all three N-donor atoms and propagating the 1D coordination polymer. The two lateral 1D chains are linked to the central one by alternating coordination (above and below the central chain) to Cu(I) by molecules of **2** (Bi1 atom in Figure 6) using just one of the three 3-Py N-donor atoms to coordinate the fourth position of the Cu center; thus, all Cu atoms are four-coordinate. The other two pyridyl arms are uncoordinated, although one of the pyridine arms is involved in a short contact with a core Bi atom. The high Lewis acidity of the Bi bridgehead atoms is manifested through anion···Bi interactions, which are particularly short for the peripheral Bi ligands [Bi···F 3.104(9) Å; cf. 3.54 Å for ∑_VDW_(Bi···F)]. The core Bi ligands also form F···Bi contacts, albeit much longer ones [3.357(9) Å], suggestive of weaker interactions.

While it is difficult to pinpoint the exact reasons for the switch in the structural type from the MOF arrangement found in the CuBF_4_ complex of **1** (**1**·CuBF_4_) and the CuPF_6_ complex of **2** (**2**·CuPF_6_) to the 1D arrangement seen in the CuBF_4_ complex of **2** (**2**·CuBF_4_), this is clearly an example of anion direction. Importantly, in this respect, the Bi(III) bridgehead of ligand **2** is significantly more Lewis acidic than the Sb(III) bridgehead of **1**, leading to an increased ability for anion–bridgehead interactions. In short, the lower Lewis basicity of the Bi center leads to weaker Bi–metal bonding and stronger anion–Bi interactions, which, if sufficiently strong, can divert the thermodynamics away from the MOF structure.

**Figure 7:**
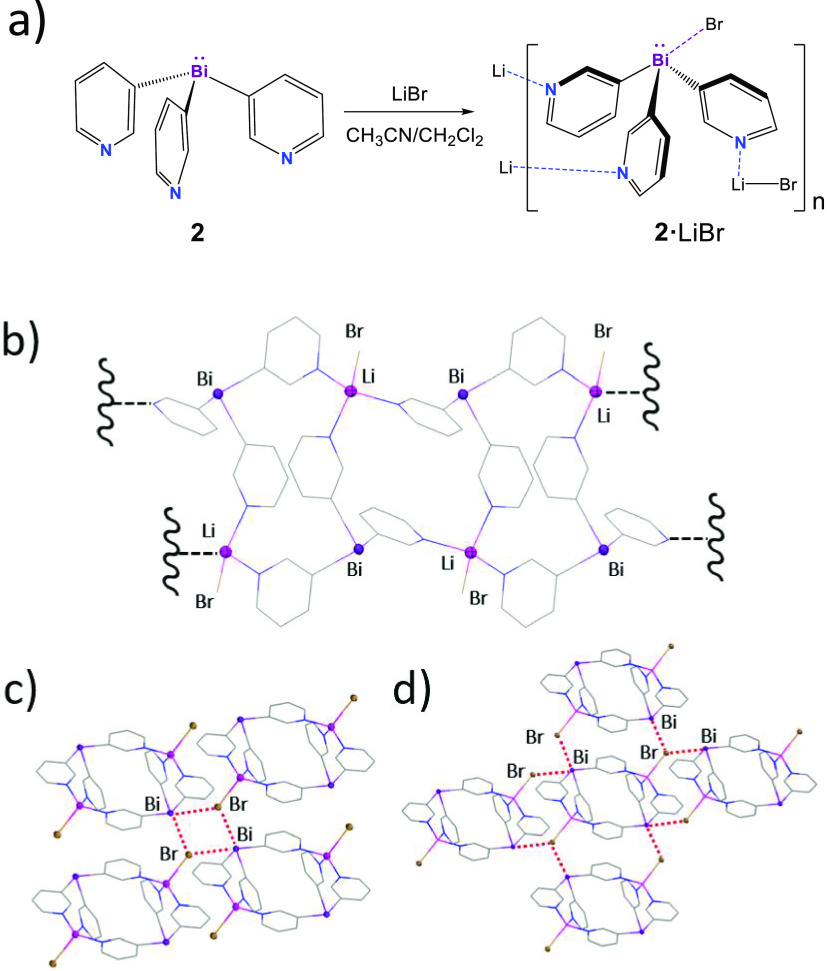
(a) Synthesis of **2**·LiBr·MeCN. (b) X-ray structure of the 1D polymeric structure of **2**·LiBr·MeCN, which is composed of fused Li_2_Bi_2_ rings. (c) View along the *b* axis and formation of the polymeric 3D structure resulting in short Bi–Br interactions between adjacent chains (d). Displacement ellipsoids at 50% probability. H atoms and one molecule of MeCN present in the asymmetric unit are omitted for clarity. Selected bond lengths (Å) and angles (deg): Bi–C_py_ 2.256(4)–2.286(3); Li–N 2.050(7)–2.075(5); Li–Br 2.517(7); C_py_–Bi–C_py_ 91.8(1)–92.9(1); N–Li–N 104.3(3)–116.3(3). Color key: Br, brown; C, gray; Li, pink; Bi, purple; N, blue.

To explore the effect of the presence of more strongly coordinating anions, we studied the reactions of **2** with CuBr and LiBr. Reaction with CuBr led to the formation of a yellow solid that was highly insoluble, precluding characterization. However, PXRD studies indicated a different outcome from that of the 3D MOFs discussed above (see the SI). LiBr was selected to explore the switch of the bridgehead from cation coordination to anion interaction. We reasoned that the hard Li^+^ would suppress bridgehead coordination of the metal ion, while the strongly coordinating Br^–^ would promote anion interaction, thus promoting N,N,N coordination to Li^+^ and Bi–Br coordination. The slow diffusion of a solution of LiBr (1 equiv) in MeCN into a DCM solution of **2** (1 equiv) over 1 week gave colorless crystals of [Bi(3-py)_3_(LiBr)(MeCN)]_*n*_ (**2**·LiBr·MeCN; Figure 7). The X-ray structure consists of a 1D coordination polymer in which the bismuthine ligand **2** uses all three pyridine donor atoms to coordinate neighboring LiBr units, resulting in a distorted four-coordinate geometry for the Li atom. This leads to the formation of a ribbonlike polymeric arrangement composed of fused Li_2_Bi_2_ rings (Figure 7a). The Lewis acidity of the bridgehead is expressed in the formation of short intermolecular Bi···Br contacts [Bi···Br 3.7272(5) and 3.6922(4) Å; cf. 3.9 Å for ∑_VDW_(Bi···Br)] of all of the Bi(III) centers of the 1D chains, illustrating the amphiphilic character of the bismuthine ligand **2**. Each Bi center interacts with two Br centers of two adjacent ribbons. An additional Bi atom completes a Bi_2_Br_2_ core (Figure 7b) linking a total of four ribbons; thus, each ribbon is engaged in a 3D structure surrounded by four ribbons (Figure 7c).

## Conclusions

In conclusion, the tris(3-pyridyl) ligands E(3-py)_3_ [E = Sb (**1**), Bi (**2**)] form a common hybrid 3D MOF arrangement with an extensive range of Cu(I) and Ag(I) salts (containing different anions X), composed of a cationic substructure of E_4_M_4_ cage constituents linked together by E–metal (M) bonding. This suggests that the ligands themselves and their ability to function as tetrahedral N,N,N,E-bonding nodes have a major structure-directing influence. However, the stability of this MOF arrangement is subtly influenced by the balance between the Lewis basicity and acidity of the bridgehead atoms (E). In the case of the bismuthine ligand **2**, the low accessibility of the lone pair on the Bi bridgehead results in inherently weak Bi–M bonds and higher Lewis acidity than that in the stibene **1**. This means that anion···bridgehead interactions can compete with metal coordination, leading to collapse of the MOF structure in the case of complexes of **2** (as seen in **2**·CuBF_4_ or **2**·LiBr·MeCN). This work underlines how subtle electronic effects of the bridgehead can modulate the coordination behavior of these ligands and provides a step forward in both understanding and directing the formation of supramolecular complexes in this area.

## Experimental Section

### General Experimental Techniques

All syntheses were carried out on a vacuum line under a N_2_ atmosphere. Products were isolated and handled under a N_2_ atmosphere. 3-Bromopyridine, NMR solvents, and reaction solvents were stored over molecular sieves and degassed using three freeze–pump–thaw cycles under N_2_ prior to use. NMR spectra were recorded using a 500 MHz Agilent DD2 instrument equipped with a cold probe and a 400 MHz Agilent instrument equipped with a ONEPROBE in the Laboratory of Instrumental Techniques (LTI) Research Facilities, University of Valladolid. Chemical shifts (δ) are reported in parts per million (ppm). ^1^H and ^13^C NMR are referenced to tetramethylsilane. Coupling constants (*J*) are reported in hertz. Standard abbreviations are used to indicate multiplicity: s = singlet, d = doublet, t = triplet, and m = multiplet. ^1^H and ^13^C peak assignments were performed with the help of additional 2D NMR experiments (^1^H–^13^C HSQC and ^1^H–^13^C HMBC). High-resolution mass spectrometry (HR-MS) spectra were recorded at the mass spectrometry service of the Centros de Apoyo a la Investigación of the University of Alcalá and the LTI of the University of Valladolid. An Agilent TOF-LC/MS 6210 spectrometer [electrospray ionization time-of-flight (ESI-TOF), positive-ion mode], a UPLC–MS system [ultraperformance liquid chromatography (UPLC), Waters ACQUITY H-class ultraperformance liquid chromatograph; MS, Bruker Maxis Impact spectrometer) by ESI (positive-ion mode), a matrix-assisted laser desorption/ionization time-of-flight (MALDI-TOF) system, and a Bruker autoflex speed (N_2_ laser: 337 nm, pulse energy = 100 μJ, 1 ns, acceleration voltage = 19 kV, reflector positive mode) were used. *trans*-2-[3-(4-*tert*-Butylphenyl)-2-methyl-2-propenylidene]malonitrile was used as the matrix. Luminescence spectra were recorded at the University of Valladolid and the Parque Científico Tecnológico (PCT) facilities of the University of Burgos. A PerkinElmer LS-55 luminescence spectrometer and an Edinburgh FLS980 fluorescence spectrometer equipped with an integrating sphere module and a single-photon photomultiplier-tube detector were used. The emission lifetime of the solid samples was carried out with time-correlated single photon counting of the FLS980 spectrometer. All data were measured at 25 °C. PXRD measurements were performed in the LTI of the University of Valladolid using a Bruker Discover D8 diffractometer. The PXRD patterns of all of the isolated compounds were coincident with those predicted by the software package *MERCURY* from single-crystal X-ray analysis (see the Experimental Section), thus confirming the identity of the bulk products.

### X-ray Diffraction Studies

Diffraction data were collected using an Oxford Diffraction Supernova diffractometer equipped with an Atlas CCD area detector and a four-circle κ goniometer. For the data collection, a Mo-microfocused source with multilayer optics was used. When necessary, crystals were mounted directly from solution using perfluorohydrocarbon oil to prevent atmospheric oxidation, hydrolysis, and solvent loss. Data integration, scaling, and empirical absorption correction were performed using the *CrysAlisPro* software package.^52^ The structure was solved by direct methods and refined by full-matrix least squares against *F*^2^ with *SHELX*(53) in *OLEX2*. Non-H atoms were refined anisotropically, and H atoms were placed at idealized positions and refined using the riding model. Graphics were made with *OLEX2*(54) and *MERCURY*.^55^ In the case of **2**·CuBF_4_ and **2**·LiBr·MeCN, the data were collected using a D8 VENTURE dual-source configuration diffractometer with an a IμS 3.0 microfocus source of Mo by the staff at the PCT, University of Burgos. For measurement of the voids of the cages, first, the anion located in the cage was removed from the X-ray structure. Once the anion was removed, the void volume (Å^3^) values for the MOFs were calculated using the “Calculate solvent accessible voids” tool in *OLEX2* using a probe radius of 1.2 Å and a grid spacing of 0.1 Å.^54^

### Computational Details

All computations were carried out using the *Gaussian16* package,^56^ in which the hybrid method of Austin, Petersson, and Frisch with spherical atom dispersion terms (APFD) was applied.^57^ The cc-pvtz-pp basis set was used for the Ag, Cu, Bi, and Sb atoms,^58−61^ as found in the EMSL basis set exchange Web site,^62^ and the double-ζ 6-31G(d′) basis set with polarization functions was used for the rest of the atoms. Geometry optimizations were performed without any symmetry restrictions, using the initial coordinates derived from the X-ray data when available, and frequency analyses were performed to ensure that a minimum structure with no imaginary frequencies was achieved in each case. NBO analysis was carried out using the program *NBO 7.0.10*(63) and EDA with the help of the *AOMIX* program.^51,64^ Visualization of the calculation results was performed using *GaussView 6.1*.^65^

#### Synthesis of **1**

3-Bromopyridine (0.96 mL, 10 mmol) was added dropwise over 30 min to a stirred solution of a 1.3 M isopropylmagnesium chloride/lithium chloride complex in THF (7.7 mL, 10 mmol, 1.3 M in THF) at −0 °C. The resulting orange solution was stirred for 1 h at 0 °C. A solution of SbBr_3_ (1.20 g, 3.33 mmol) in THF (10 mL) was added dropwise to the orange solution. The resulting mixture was allowed to reach room temperature. After overnight stirring, a colorless solution with a white precipitate was observed. All volatiles were removed under vacuum. DCM (100 mL) was added to the precipitate, and the resulting white suspension was treated with H_2_O (50 mL × 3). The organic phase was dried over anhydrous magnesium sulfate. The suspension was filtered through Celite to yield a colorless solution, which was concentrated under vacuum, and slow diffusion of *n*-hexane (20 mL) at −24 °C yielded **1** as white plates suitable for X-ray crystallography. Yield: 0.87 g (2.44 mmol, 74%). ^1^H NMR (298 K, CDCl_3_, 500 MHz): δ 8.61 (m, 6H, H_2_ + H_6_py), 7.69 (dt, *J* = 1.8/7.7 Hz, 3H, H_4_py), 7.30 (m, 3H, H_5_py). ^13^C{^1^H} NMR (298 K, CDCl_3_, 100.6 MHz): δ 156.06 (C_2_py), 150.57 (C_6_py), 143.60 (C_4_py), 131.95 (C_3_py), 125.00 (C_5_py). Elem anal. Calcd for **1** (C_15_H_12_N_3_Sb): C, 50.6; H, 3.4; N, 11.8. Found: C. 50.3; H. 3.5; N. 12.1. HR-MS (MALDI, positive-ion mode MALDI-TOF). Calcd for C_15_H_13_N_3_Sb ([**1** + H]^+^): *m*/*z* 356.0142. Found: *m*/*z* 356.0141 (−0.1 ppm error).

#### Synthesis of **2**

3-Bromopyridine (1.92 mL, 20 mmol) was added dropwise over 30 min to a stirred solution of a 1.3 M isopropylmagnesium chloride/lithium chloride complex in THF (15.4 mL, 20 mmol, 1.3 M in THF) at −0 °C. The resulting orange solution was stirred for 1 h at 0 °C. A solution of BiBr_3_ (2.99 g, 6.66 mmol) in THF (10 mL) was added dropwise to the orange solution. The resulting mixture was allowed to reach room temperature. After overnight stirring, a colorless solution with a white precipitate was observed. All volatiles were removed under vacuum. DCM (200 mL) was added to the precipitate, and the resulting white suspension was treated with H_2_O (100 mL × 3). The organic phase was dried over anhydrous magnesium sulfate. The suspension was filtered through Celite to yield a colorless solution, which was concentrated under vacuum, and slow diffusion of *n*-hexane (20 mL) at −24 °C yielded **2** as white plates suitable for X-ray crystallography. Yield: 2.38 g (5.36 mmol, 80%). ^1^H NMR (298 K, CDCl_3_, 500 MHz): δ 8.76 (s, 3H, H_2_py), 8.64 (d, *J* = 5.0 Hz, 3H, H_6_py), 8.02 (d, *J* = 7.6 Hz, 3H, H_4_py), 7.34 (m, 3H, H_5_py). ^13^C{^1^H} NMR (298 K, CDCl_3_, 100.6 MHz): δ 157.05 (C_2_py), 149.55 (C_6_py), 147.82 (br, C_3_py), 144.91 (C_4_py), 126.57 (C_5_py). Elem anal. Calcd for **2** (C_15_H_12_N_3_Bi): C, 40.6; H, 2.7; N, 9.5. Found: C, 40.4; H, 2.8; N, 9.5. HR-MS [ESI, positive-ion mode ESI-TOF]. Calcd for C_15_H_13_N_3_Bi ([**2** + H]^+^): *m*/*z* 444.0908. Found: *m*/*z* 444.0922 (−1.4 ppm error).

#### Synthesis of **1**·CuBF_4_

A solution of **1** (35 mg, 0.098 mmol) in dry DCM (5 mL) was prepared in a narrow Schlenk flask under a N_2_ atmosphere. A layer of MeCN (5 mL) was layered on top of the ligand solution, and a solution of [Cu(MeCN)_4_]BF_4_ (30.9 mg, 0.098 mmol) in dry MeCN (5 mL) was then layered carefully on top of the MeCN layer. The three layers were left to diffuse slowly at room temperature over 1 week, resulting in yellow crystals of **1**·CuBF_4_ suitable for X-ray crystallography. Yield: 33 mg (0.071 mmol, 72%). Characterization of the product by NMR spectroscopy and mass spectrometry was impeded by its insolubility in suitable solvents. Elem anal. Calcd for **1**·CuBF_4_ (C_15_H_12_NCuF_4_N_3_Sb): C, 35.6; H, 2.4; N, 8.3. Found: C, 35.5; H, 2.5; N, 8.5. In addition, the precipitate and crystals were confirmed to be identical by PXRD (see the SI).

#### Synthesis of **1**·CuPF_6_

A solution of **1** (25 mg, 0.070 mmol) in dry DCM (5 mL) was prepared in a narrow Schlenk flask under a N_2_ atmosphere. A layer of MeCN (5 mL) was layered on top of the ligand solution, and a solution of [Cu(MeCN)_4_]PF_6_ (26.2 mg, 0.070 mmol) in dry MeCN (5 mL) was then layered carefully on top of the MeCN layer. The three layers were left to diffuse slowly at room temperature for a week, resulting in yellow crystals of **1**•CuPF_6_ suitable for X-ray crystallography. Yield: 31.2 mg (0.055 mmol, 79%). Characterization of the product by NMR spectroscopy and MS was impeded by its insolubility in suitable solvents. Elem anal. Calcd for **1**·CuPF_6_ (C_15_H_12_CuF_6_N_3_PSb): C, 31.9; H, 2.1; N, 7.4. Found: C, 32.1; H, 2.4; N, 7.7. In addition, the precipitate and the crystals were confirmed to be identical by powder diffraction (see the SI).

#### Synthesis of **1**·AgBF_4_

A solution of **1** (40 mg, 0.11 mmol) in dry DCM (5 mL) was prepared in a narrow Schlenk flask under a N_2_ atmosphere in the dark. A layer of MeCN (5 mL) was layered on top of the ligand solution, and a solution of AgBF_4_ (21.9 mg, 0.11 mmol) in dry MeCN (5 mL) was then layered carefully on top of the MeCN layer. The three layers were left to diffuse slowly at room temperature for 1 week, resulting in colorless crystals of **1**·AgBF_4_suitable for X-ray crystallography. Yield: 35 mg (0.064 mmol, 58%). Characterization of the product by NMR spectroscopy was impeded by its insolubility in suitable solvents. Elem anal. Calcd for **1**·AgBF_4_ (C_15_H_12_AgBF_4_N_3_Sb): C, 32.7; H, 2.2; N, 7.6. Found: C, 32.7; H, 2.3; N, 7.7. HR-MS [ESI, positive-ion mode ESI-TOF]. Calcd for C_15_H_12_AgN_3_Sb ([**1** + Ag]^+^): *m*/*z* 463.9115. Found: *m*/*z* 463.9125 (−1.0 ppm error). In addition, the precipitate and crystals were confirmed to be identical by PXRD (see the SI).

#### Synthesis of **1**·AgPF_6_

A solution of **1** (25 mg, 0.070 mmol) in dry DCM (5 mL) was prepared in a narrow Schlenk flask under a N_2_ atmosphere in the dark. A layer of MeCN (5 mL) was layered on top of the ligand solution, and a solution of AgPF_6_ (17.7 mg, 0.070 mmol) in dry MeCN (5 mL) was then layered carefully on top of the MeCN layer. The three layers were left to diffuse slowly at room temperature for 1 week, resulting in colorless crystals of **1**·AgPF_6_ suitable for X-ray crystallography. Yield: 22.3 mg (0.036 mmol, 52%). Characterization of the product by NMR spectroscopy and MS was impeded by its insolubility in suitable solvents. Elem anal. Calcd for **1**·AgPF_6_ (C_15_H_12_AgF_6_N_3_PSb): C, 29.6; H, 2.0; N, 6.9. Found: C, 29.5; H, 1.2; N, 7.0. In addition, the precipitate and crystals were confirmed to be identical by PXRD (see the SI).

#### Synthesis of **1**·AgSbF_6_

A solution of **1** (40 mg, 0.11 mmol) in dry DCM (5 mL) was prepared in a narrow Schlenk flask under a N_2_ atmosphere in the dark. A layer of MeCN (5 mL) was layered on top of the ligand solution, and then a solution of AgSbF_6_ (38.5 mg, 0.11 mmol) in dry MeCN (5 mL) was layered carefully on top of the MeCN layer. The three layers were left to diffuse slowly at room temperature over 1 week, resulting in colorless crystals of **1**·AgSbF_6_ suitable for X-ray crystallography. Yield: 38.6 mg (0.057 mmol, 51%). Characterization of the product by NMR spectroscopy and MS was impeded by its insolubility in suitable solvents. Elem anal. Calcd for **1**·AgSbF_6_ (C_15_H_12_AgF_6_N_3_Sb_2_): C, 25.8; H, 1.7; N, 6.0. Found: C, 26.0; H, 1.9; N, 6.5. In addition, the precipitate and crystals were confirmed to be identical by PXRD (see the SI).

#### Synthesis of **1**·AgOTf

A solution of **1** (25 mg, 0.070 mmol) in dry DCM (5 mL) was prepared in a narrow Schlenk flask under a N_2_ atmosphere in the dark. A layer of MeCN (5 mL) was layered on top of the ligand solution, and then a solution of AgOTf (18 mg, 0.070 mmol) in dry MeCN (5 mL) was layered carefully on top of the MeCN layer. The three layers were left to diffuse slowly at room temperature over 1 week, resulting in colorless crystals of **1**·AgOTf suitable for X-ray crystallography. Yield: 25 mg (0.041 mmol, 59%). Characterization of the product by NMR spectroscopy and MS was impeded by its insolubility in suitable solvents. Elem anal. Calcd for **1**·AgOTf (C_16_H_12_AgF_3_N_3_O_3_SSb): C, 31.4; H, 2.0; N, 6.9; S, 5.2. Found: C, 31.6; H, 2.0; N, 7.5; S, 5.3. In addition, the precipitate and crystals were confirmed to be identical by PXRD (see the SI).

#### Synthesis of **2**·CuPF_6_

A solution of **2** (50 mg, 0.11 mmol) in dry DCM (5 mL) was prepared in a narrow Schlenk flask under a N_2_ atmosphere. A layer of MeCN (5 mL) was layered on top of the ligand solution, and a solution of [Cu(MeCN)_4_]PF_6_ (42 mg, 0.11 mmol) in dry MeCN (5 mL) was then layered carefully on top of the MeCN layer. The three layers were left to diffuse slowly at room temperature for 1 month, resulting in yellow crystals of **2**·CuPF_6_ suitable for X-ray crystallography. Yield: 34.3 mg (0.052 mmol, 48%). Characterization of the product by NMR spectroscopy and MS was impeded by its insolubility in suitable solvents. Elem anal. Calcd for **2**·CuPF_6_ (C_15_H_12_BiCuF_6_N_3_P): C, 27.6; H, 1.9; N, 6.4. Found: C, 27.9; H, 2.1; N, 6.3. In addition, the precipitate and crystals were confirmed to be identical by PXRD (see the SI).

#### Synthesis of **2**·AgSbF_6_

A solution of **2** (40 mg, 0.090 mmol) in dry DCM (5 mL) was prepared in a narrow Schlenk flask under a N_2_ atmosphere in the dark. A layer of MeCN (5 mL) was layered on top of the ligand solution, and a solution of AgSbF_6_ (31.3 mg, 0.090 mmol) in dry MeCN (5 mL) was then layered carefully on top of the MeCN layer. The three layers were left to diffuse slowly at room temperature for 1 month, resulting in colorless crystals of **2**·AgSbF_6_ suitable for X-ray crystallography. Yield: 22.5 mg (0.028 mmol, 32%). Characterization of the product by NMR spectroscopy and MS was impeded by its insolubility in suitable solvents. Elem anal. Calcd for **2**·AgSbF_6_ (C_15_H_12_AgBiF_6_N_3_Sb): C, 22.9; H, 1.5; N, 5.3. Found: C, 21.3; H, 0.5; N, 5.1. The crystals of **2**·AgSbF_6_ were isolated in the presence of a black amorphous solid, which hampered elemental analysis. In addition, the precipitate and crystals were confirmed to be identical by PXRD (see the SI).

#### Synthesis of **2**·CuBF_4_

A solution of **2** (30 mg, 0.067 mmol) in dry DCM (5 mL) was prepared in a narrow Schlenk flask under a N_2_ atmosphere. A solution of [Cu(MeCN)_4_]BF_4_ (10.5 mg, 0.037 mmol) in dry MeCN (5 mL) was layered carefully on top of the ligand solution. The two layers were left to diffuse slowly at room temperature for 1 month, resulting in yellow crystals of **2**·CuBF_4_ suitable for X-ray crystallography. Yield: 14.3 mg (0.013 mmol, 37.3%). Characterization of the product by NMR spectroscopy and MS was impeded by its insolubility in suitable solvents. Elem anal. Calcd for **2**·CuBF_4_ (C_30_H_24_BBi_2_CuF_4_N_6_): C, 34.7; H, 2.3; N, 8.1. Found: C, 34.2; H, 2.4; N, 8.2. In addition, the precipitate and crystals were confirmed to be identical by PXRD (see the SI).

#### Synthesis of **2**·LiBr·MeCN

A solution of **2** (50 mg, 0.11 mmol) in dry DCM (5 mL) was prepared in a narrow Schlenk flask under a N_2_ atmosphere. A solution of LiBr (9.8 mg, 0.11 mmol) in dry MeCN (5 mL) was layered carefully on top of the ligand solution. The two layers were left to diffuse slowly at room temperature over 1 month, resulting in yellow crystals of **2**·LiBr·MeCN suitable for X-ray crystallography. Yield: 30.2 mg (0.052 mmol, 48%). Characterization of the product by NMR spectroscopy and MS was impeded by its insolubility in suitable solvents. Elem anal. Calcd for **2**·LiBr·MeCN (C_17_H_15_BiBrLiN_4_): C, 35.7; H, 2.6; N, 9.8. Found: C, 35.0; H, 2.7; N, 9.7. In addition, the precipitate and crystals were confirmed to be identical by PXRD (see the SI).

## Supporting Information Available

NMR spectra, additional X-ray structures, details and information, and HR-MS data (PDF)

## Accession Codes

CCDC 2110782–2110793 contain the supplementary crystallographic data for this paper. These data can be obtained free of charge via www.ccdc.cam.ac.uk/data_request/cif, or by emailing data_request@ccdc.cam.ac.uk, or by contacting The Cambridge Crystallographic Data Centre, 12 Union Road, Cambridge CB2 1EZ, UK; fax: +44 1223 336033.
