# Supplementary material for: Cation- and Anion-Mediated Supramolecular Assembly of Bismuth and Antimony Tris(3-pyridyl) Complexes
Source: Inorg Chem. 2021 Dec 9;60(24):19206–18. doi: 10.1021/acs.inorgchem.1c03004 (PMC8693195; doi:10.1021/acs.inorgchem.1c03004)
Supplement: Supplementary file 1 — ic1c03004_si_001.pdf [file ic1c03004_si_001.pdf]

Supporting Information

Cation- and Anion-Mediated Supramolecular Assembly of Bismuth and Antimony Tris(3-pyridyl) Complexes

Álvaro García-Romero,† Jose M. Martín-Álvarez†, Daniel Miguel,† Dominic S. Wright,‡ Celedonio M. Álvarez,,† and Raúl García-Rodríguez*,†*

†GIR MIOMeT-IU, Cinquima, Química Inorgánica, Facultad de Ciencias, Universidad de Valladolid, Campus Miguel Delibes, 47011, Valladolid, Spain. E-mail: celedonio.alvarez@uva.es , raul.garcia.rodriguez@uva.es

‡Department of Chemistry, University of Cambridge, Lensfield Road, Cambridge, CB2 1EW, U.K.

Contents

NMR studies and NMR spectra **4**

*Figure S1. Compound **1**, Sb(3-py)$_3$* 4

*Figure S2. ^{1}H NMR spectrum of **1*** 4

*Figure S3. ^{13}C{^{1}H} NMR spectrum of **1*** 4

*Figure S4. ^{1}H–^{13}C HSQC spectrum of **1*** 5

*Figure S5. Selected region of the ^{1}H–^{13}C HMBC spectrum of **1*** 5

*Figure S6. Compound **2**, Bi(3-py)$_3$* 6

*Figure S7. ^{1}H NMR spectrum of **2*** 6

*Figure S8. ^{13}C{^{1}H} NMR spectrum of **2*** 6

*Figure S9. Stacked ^{1}H NMR spectra of **1** with THTAuCl* 7

*Figure S10. Stacked ^{1}H NMR spectra of **2** with THTAuCl* 7

High resolution mass data **8**

*Figure S11. HR-MS (MALDI-TOF) of **1*** 8

*Figure S12. HR-MS (ESI-TOF) of **2*** 8

*Figure S13. HR-MS (ESI-TOF) of (**1**•AgBF$_4$)* 9

Figure S14. HR-MS (ESI-TOF) of 3,3´-bipyridine 9

X-ray Crystallographic Studies **10**

Figure S15. Three-layer synthesis method to obtain crystals for X-ray diffraction studies 10

Details of the refinement methods of the structures 10

Figure S16. Compounds 1•CuBF$_4$ and 1•CuPF$_6$ 11

Figure S17. Compounds 1•AgBF$_4$, 1•AgPF$_6$, 1•AgSbF$_6$ and 1•AgOTf 12

Figure S18. Compounds 2•CuPF$_6$ and 2•AgSbF$_6$ 13

Figure S19. Values for the angle N–M–Sb for the Sb and Bi MOFs 14

Figure S20. Values of ΣM_α for the Sb and Bi MOFs 14

Table S1. Void volumes of the cage units 15

Figure S21. Representation of the the void volumes the Sb MOFs 15

Figure S22. Linear relationship observed between the cage volume and anion volume 16

Table S2. Crystallographic data 17

Table S3. Selected bond length and angles 20

Powder X-ray studies **21**

*Figure S23. Comparison of the experimental and the predicted XRPD patterns for **1**•CuBF$_4$* 21

*Figure S24. Comparison of the experimental and the predictedXRPD patterns for **1**•CuPF$_6$* 21

*Figure S25. Comparison of the experimental and the predicted XRPD patterns for **1**•AgBF$_4$* 22

*Figure S26. Comparison of the experimental and the predicted XRPD patterns for **1**•AgPF$_6$* 22

*Figure S27. Comparison of the experimental and the predicted XRPD patterns for **1**•AgSbF$_6$* 23

*Figure S28. Comparison of the experimental and the predicted XRPD patterns for **1**•AgOTf*.23

*Figure S29. Comparison of the experimental and the predicted XRPD patterns for **2**•$CuPF_6$* .24

*Figure S30. Comparison of the experimental and the predicted XRPD patterns for **2**•$AgSbF_6$*24

*Figure S31. Comparison of the experimental and the predicted XRPD patterns for **2**•$CuBF_4$* .25

*Figure S32. Comparison of the experimental and the predicted XRPD patterns for **2**•LiBr*.....25

*Figure S33. Experimental XRPD pattern for the yellow insoluble solid obtained from the reaction of equimolar amounts of **2** and CuBr* 26

Emission studies for selected copper complexes 27

*Figure S34. Emission spectra of **1**•$CuPF_6$ and **2**•$CuPF_6$*...27

*Figure S35. Emission spectra of **1**•$CuPF_6$, **1**•$CuBF_4$ and **2**•$CuPF_6$.* 27

*Table S4. Excitation and emission data (nm) for crystal powders of **1**•$CuPF_6$ and **2**•$CuPF_6$.*...28

*Figure S36. Exponential components analysis for **1**•$CuPF_6$* 28

*Figure S37. Exponential components analysis for **2**•$CuPF_6$* 28

Computational details..29

Table S5. Bi-Ag distance for various functionals 29

Table S6. Energy of the E-M interaction and composition of the orbital involved 29

Figure S38. Simple model of the Bi MOF 2•$AgSbF_6$ 30

References 37

NMR studies and NMR spectra

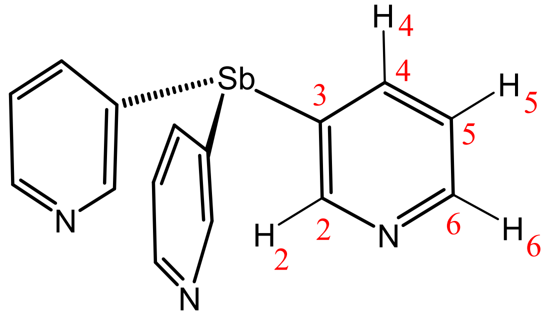

*Figure S1. Compound **1**, Sb(3-py)$_3$, with the atom labelling used in the NMR studies.*

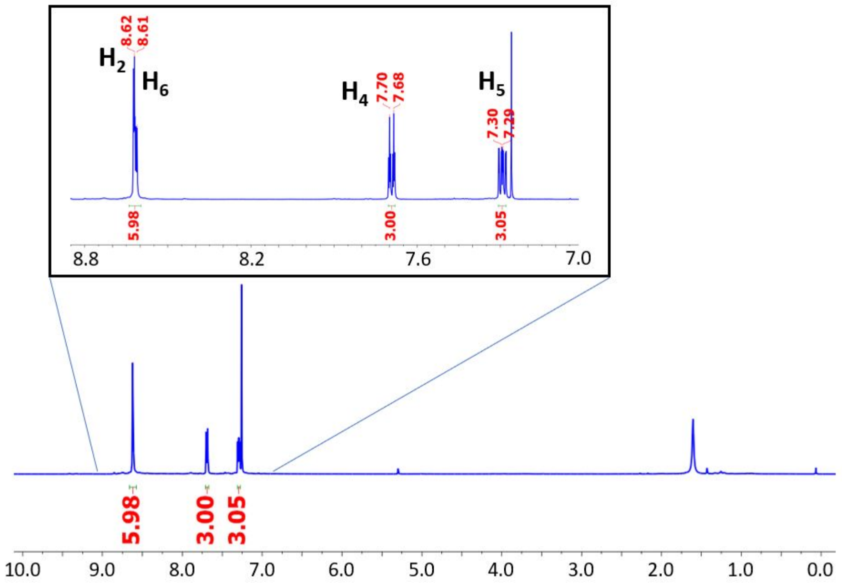

*Figure S2. ^{1}H NMR (298 K, CDCl$_3$, 500 MHz) spectrum of **1**.*

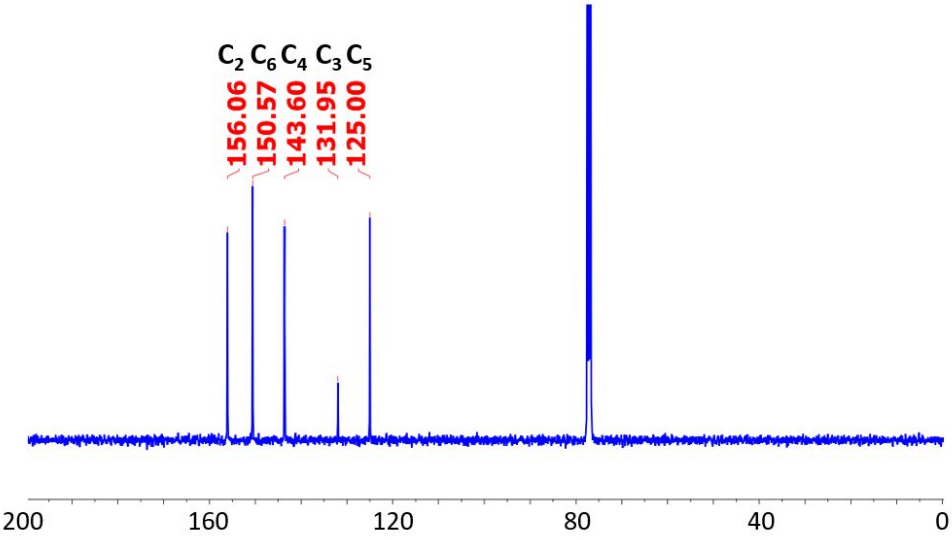

*Figure S3. ^{13}C{^{1}H} NMR (298 K, CDCl$_3$, 100.5 MHz) spectrum of **1**.*

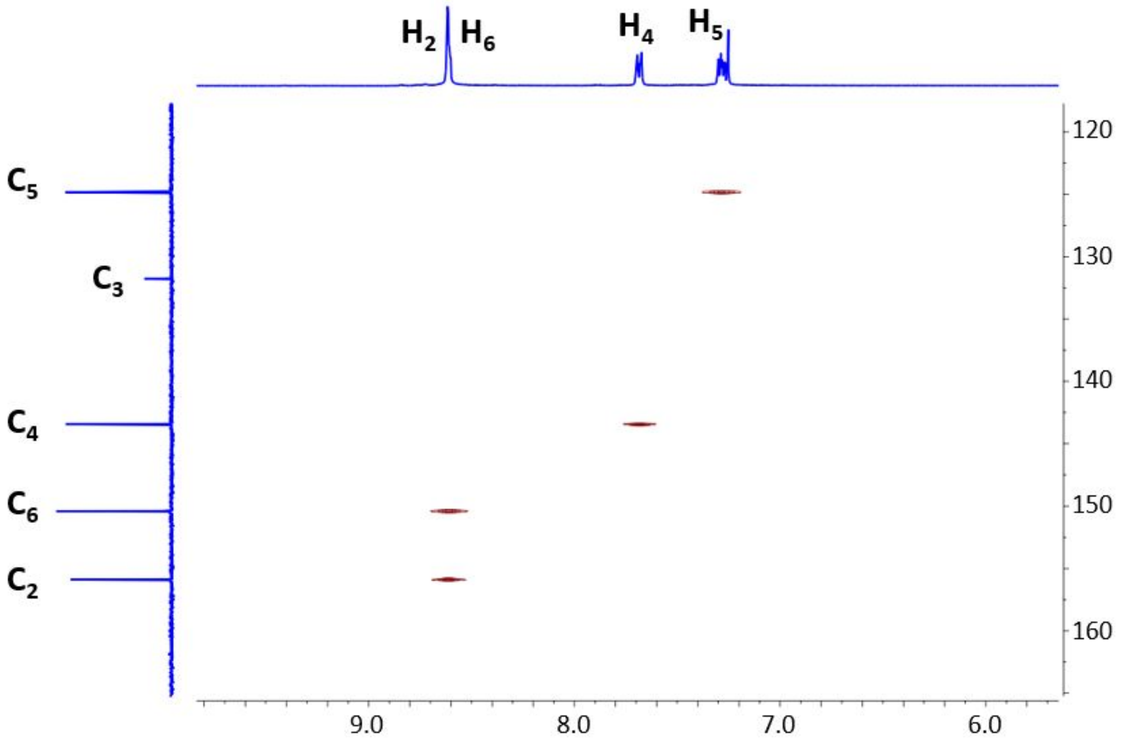

*Figure S4. ^{1}H–^{13}C HSQC (298 K, $CDCl_3$) spectrum of **1**.*

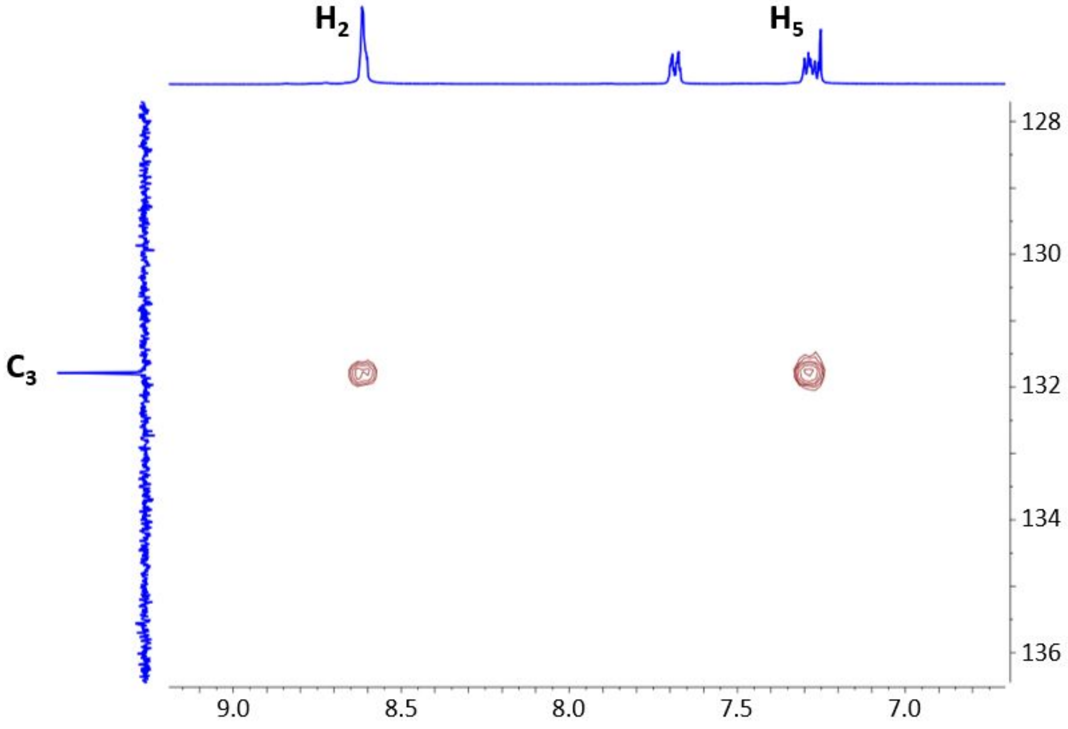

*Figure S5. Selected region of the ^{1}H–^{13}C HMBC (298 K, $CDCl_3$) spectrum of **1** used for the identification of the Sb–C bonded carbon (C_3) (signal at 131.95 ppm).*

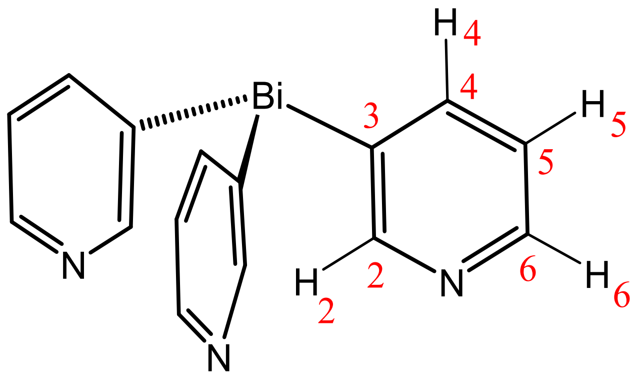

*Figure S6. Compound **2**, Bi(3-py)$_3$, with the atom labelling used in the NMR studies.*

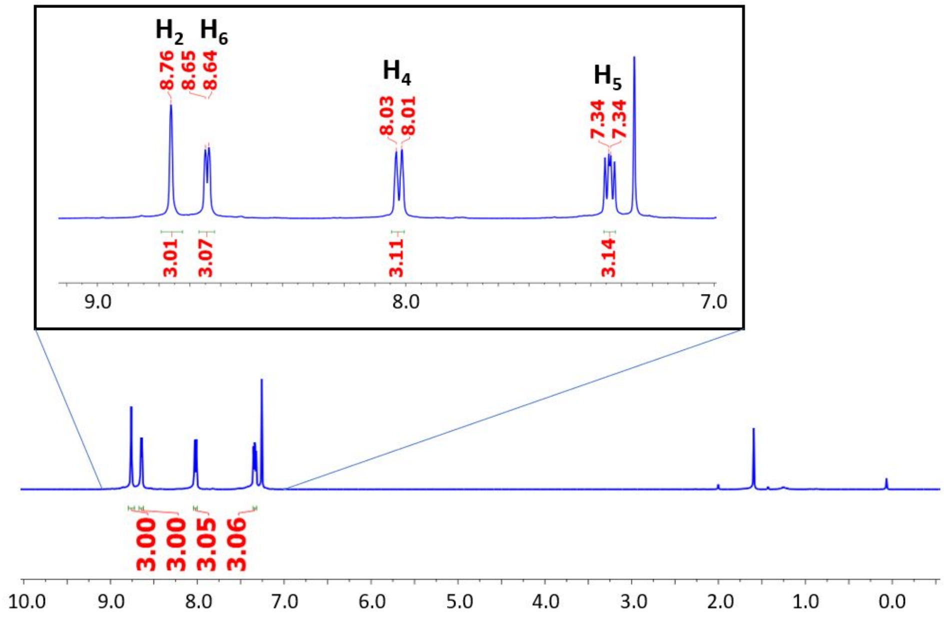

*Figure S7. ^{1}H NMR (298 K, CDCl$_3$, 500 MHz) spectrum of **2**.*

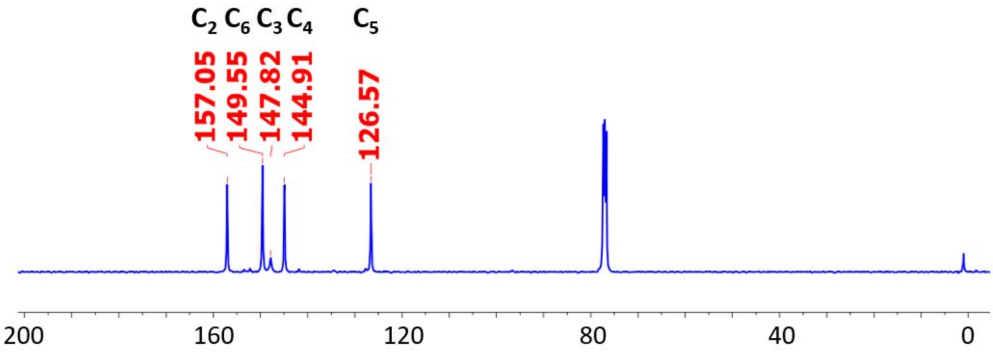

*Figure S8. ^{13}C{^{1}H} NMR (298 K, CDCl$_3$, 100.5 MHz) spectrum of **2**.*

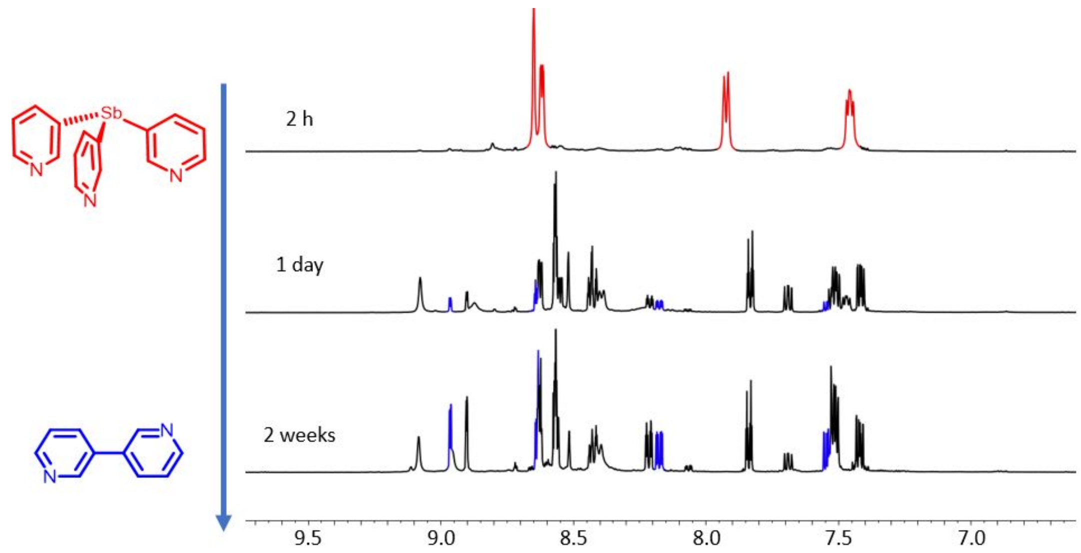

*Figure S9. Stacked ^{1}H NMR spectra of the evolution of a sample of an equimolar amount of **1** and THTAuCl in DMSO-d_6 over 2 hours, 1 day and 2 weeks at r.t. After 2 weeks the appearance of 3,3-bipyridine (in blue), along with other unidentified species, was observed.*

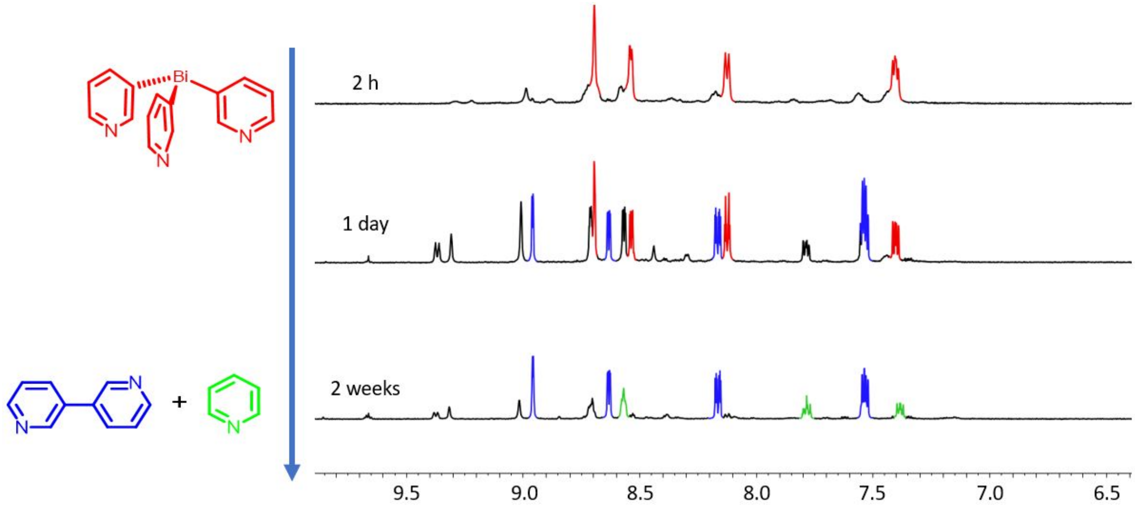

*Figure S10. Stacked ^{1}H NMR spectra of the evolution of a sample of an equimolar amount of **2** and THTAuCl in DMSO-d_6 over 2 hours, 1 day and 2 weeks at r.t. After 2 weeks the appearance of 3,3-bipyridine and free pyridine, along with small amounts of other unidentified species, was observed.*

High resolution mass data

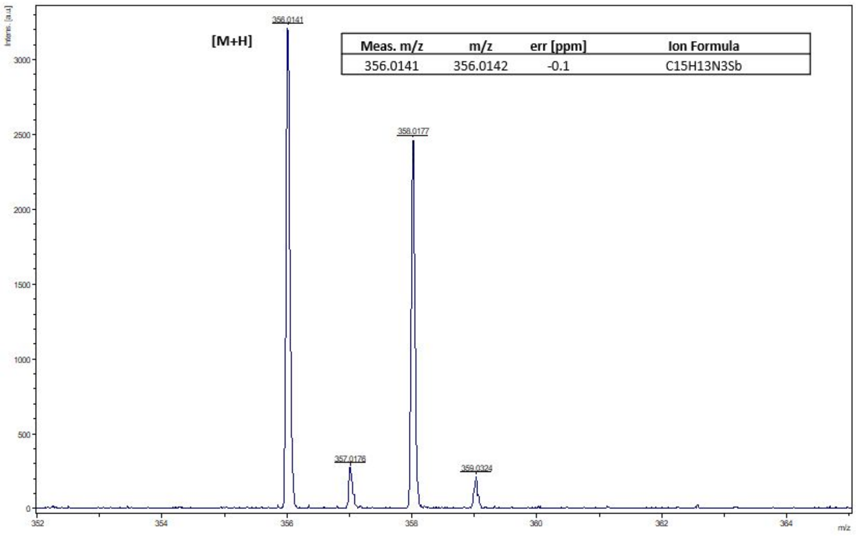

*Figure S11. HR-MS (MALDI-TOF) of **1**.*

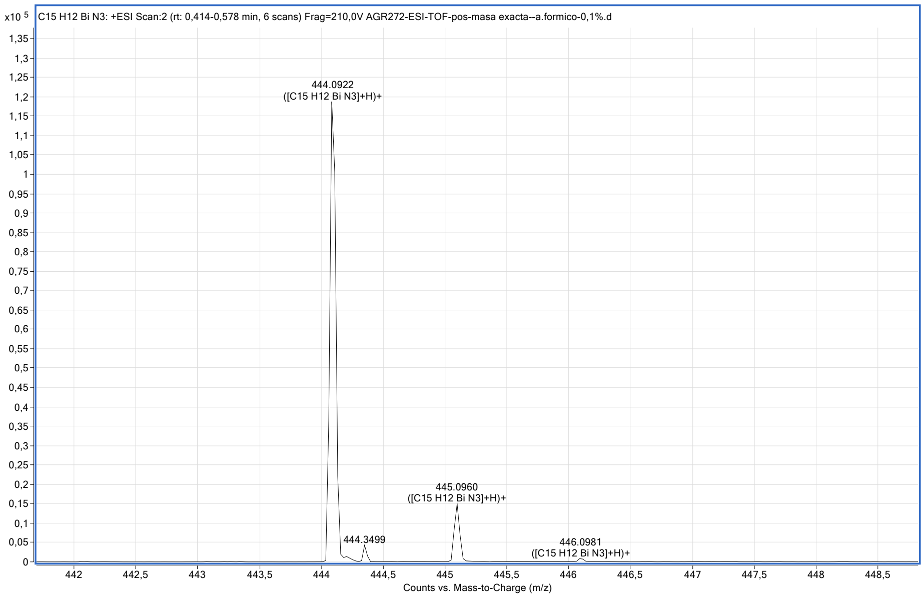

Best	ID Source	Name	Formula	Species	m/z	Score	Score (RT)	RT Diff	Diff (ppm)	Score (Lib)	Score (DB)	Score (MFG)	Nu
⦿	MFG		C15 H12 Bi N3	(M+H)+	444.0922	75,08			-3,43			75,08	

Species	m/z	Score (iso. abund)	Score (mass)	Score (MFG, MS/MS)	Score (MS)	Score (MFG)	Score (iso. spacing)	Height	Ion Formula
(M+H)+	444,0922	74,6	89,16		75,08	75,08	47,49	123864,7	C15 H13 Bi N3

Height (Calc)	Height Sum% (Calc)	Height % (Calc)	m/z (Calc)	Diff (mDa)	Height	Height %	Height Sum %	m/z	Diff (ppm)
118231,7	85,1	100	444,0908	-1,4	123864,7	100	89,2	444,0922	-3,23
20654	14,9	17,5	445,0938	-2,2	15021	12,1	10,8	445,096	-5,02

*Figure S12. HR-MS (ESI-TOF) of **2**.*

The study of the extended supramolecular complexes by mass spectrometry proved challenging and not very informative. An illustrative example is given below only for the case of **1**•$AgBF_4$, for which the fragment [**1**+Ag]$^+$ was identified.

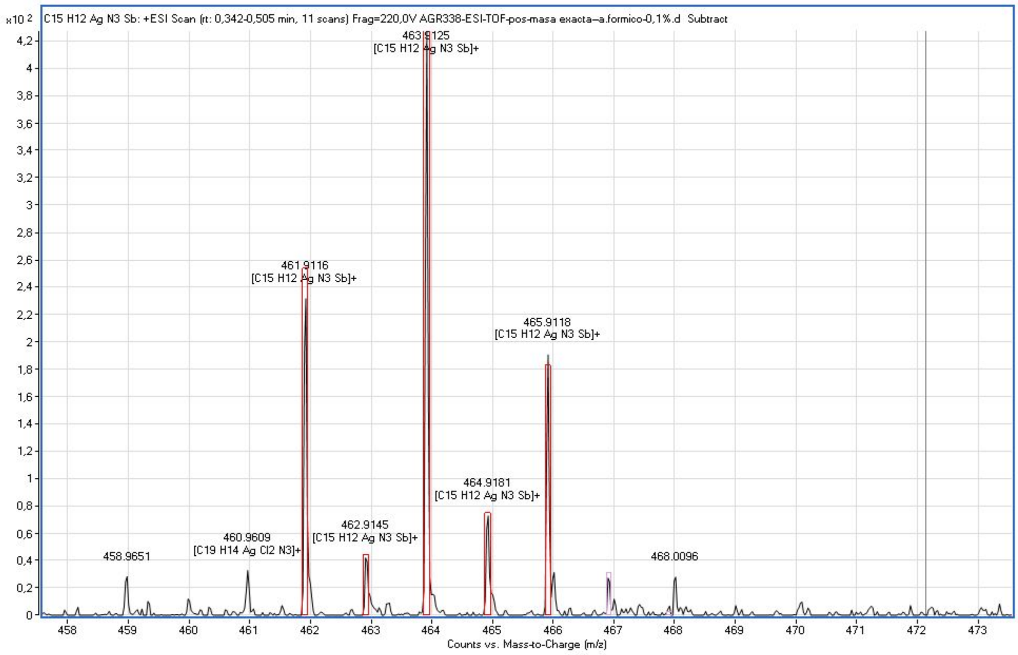

Best	ID Source	Name	Formula	Species	m/z	Score	Score (RT)	RT Diff	Diff (ppm)	Score (Lib)	Score (DB)	Score (MFG)
	MFG		C15 H12 Ag N3 Sb	M+	461.9116	92,82			-1,62			92,82

Species	m/z	Score (iso. abund)	Score (mass)	Score (MFG, MS/MS)	Score (MS)	Score (MFG)	Score (iso. spacing)	Height	Ion Formula
M+	461,9116	80,49	97,38		92,82	92,82	98,5	240,6	C15 H12 Ag N3 Sb

Height (Calc)	Height Sum% (Calc)	Height % (Calc)	m/z (Calc)	Diff (mDa)	Height	Height %	Height Sum %	m/z	Diff (ppm)
253,4	25,8	59,1	461,9115	-0,1	240,6	56,1	24,5	461,9116	-0,14
44,2	4,5	10,3	462,9145	-0,1	45,6	10,6	4,6	462,9145	-0,16
428,5	43,6	100	463,9115	-1	429,2	100	43,7	463,9125	-2,17
74,4	7,6	17,4	464,9145	-3,7	75,5	17,6	7,7	464,9181	-7,9
182,1	18,5	42,5	465,9117	0	191,7	44,7	19,5	465,9118	-0,08

*Figure S13. HR-MS (ESI-TOF) of (**1**•$AgBF_4$).*

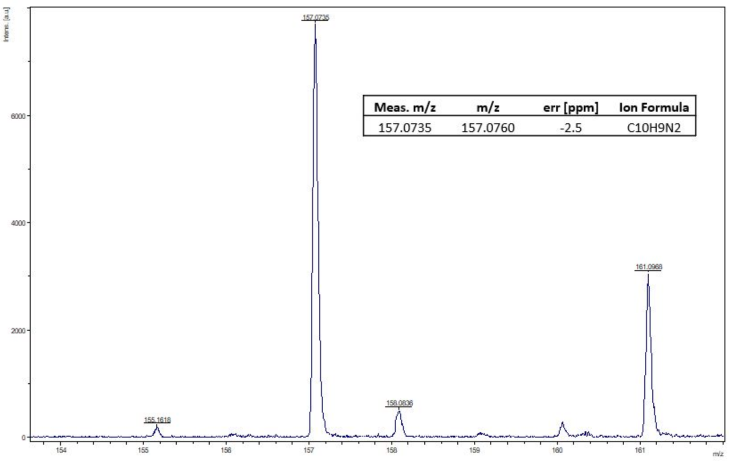

Figure S14. HR-MS (ESI-TOF) indicating the formation of 3,3´-bipyridine from the reaction crude of **1** with AuCl(THT). The identification of the formation of 3,3´-bipyridine in the reaction mixture was also done by ^{1}H NMR (see Fig S9).

X-ray Crystallographic Studies

Crystallization conditions to obtain quality crystals for single-crystal X-ray diffraction studies

As a general procedure, X-ray quality crystals were obtained by slow diffusion of a solution of the corresponding metal precursor (1 equivalent in 5 mL MeCN) into a DCM solution of **1** or **2** (typically 25–50 mg). In order to increase the quality of the crystals, the process can be slowed by placing an intermediate layer of MeCN (ca. 3 mL) between the other two to obtain a more controlled diffusion (Fig. S15). This leads to better quality crystals.

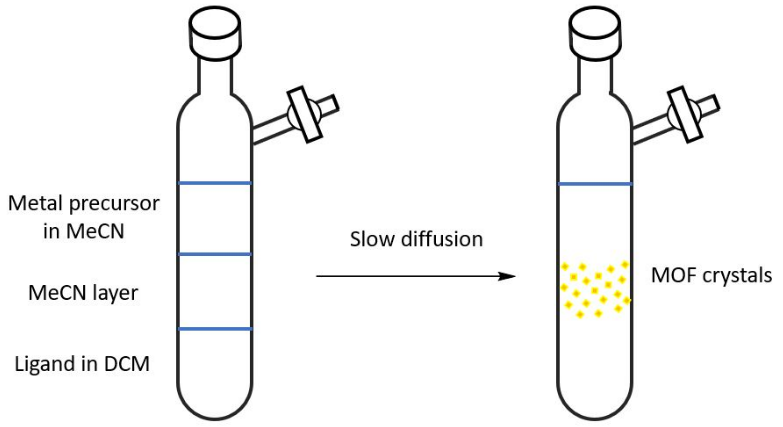

Figure S15. Three-layer synthesis method to obtain high-quality crystals for single-crystal X-ray diffraction studies.

Details of the data collections and structural refinements are given in Table S2. Further details of the methods of refinement of the structures are as follows.

1•$CuBF_4$ and **1**•$AgBF_4$**:** The BF_4^- anions were disordered and very poorly resolved (disordered by symmetry, on special positions) and they were treated as a rigid body with their site occupancies in the asymmetric unit constrained to 1/4 (for the BF_4^- in the void) and 1/12 (for the BF_4^- in the cage), to produce a total of 8 BF_4^- anions in the unit cell (2 in the cage and 6 in the exo voids, as in the related structures with PF_6^- and SbF_6^- anions discussed in the main text). The structure is charge balanced, with 8 Cu^+ and 8 BF_4^- in the unit cell. Restraints were applied to maintain sensible bond distances and geometry, and the BF_4^- anions were treated as a rigid body for the final refinement cycles. Rigu restraints were applied to keep the ADPS of the disordered BF_4^- anions to a reasonable value. The presence of BF_4^- in the crystal was also assigned on the basis of the additional characterization information, as described in the main text, including ^{19}F NMR and elemental analysis.

1•AgOTf**:** The framework structure is clear; however, the OTf⁻ anions are not clearly resolved and therefore SQUEEZE[1] was applied (i.e., removing the disordered OTF anions). SQUEEZE was applied accounting for 566 electrons in a total solvent accessible volume of 1054 cubic angstroms per unit cell. This is consistent with the presence of 8 triflates ($CF_3SO_3^-$) per unit cell (i.e., 1 CF_3SO_3 per formula unit), located in 8 voids (2 cages and 6 exo voids), which account for 592 electrons per unit cell. This is also consistent with the expected stoichiometry (i.e., one $CF_3SO_3^-$ per Ag^+ cation), as well as with the elemental analysis. The presence of OTf⁻ in the crystal was also determined by ^{19}F NMR.

X-ray diffraction structures

1•$CuBF_4$

1•$CuPF_6$

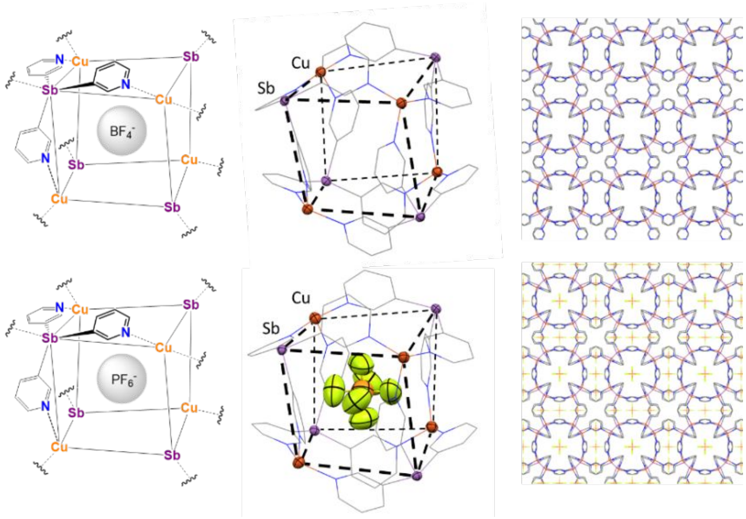

*Figure S16. Compounds 1•$CuBF_4$ and 1•$CuPF_6$: (left) Schematic representation of the formation of the monomeric cubane unit. (center) Crystal structure showing the heterocubane monomeric unit [Cu(**1**)(⊂X)]$^{3+}$(X= BF_4^- or PF_6^-) of the 3D-MOF. In the case of 1•$CuBF_4$, disordered BF_4^- anions are omitted for clarity. (right) Wireframe X-ray structure along the a, b or c axis of the 3D structure of the compound MOFs. Displacement ellipsoids at 50% probability. H atoms are omitted for clarity. Colour key: C (gray), Cu (orange), P (light orange), Sb (light purple), N (blue) and F (yellow).*

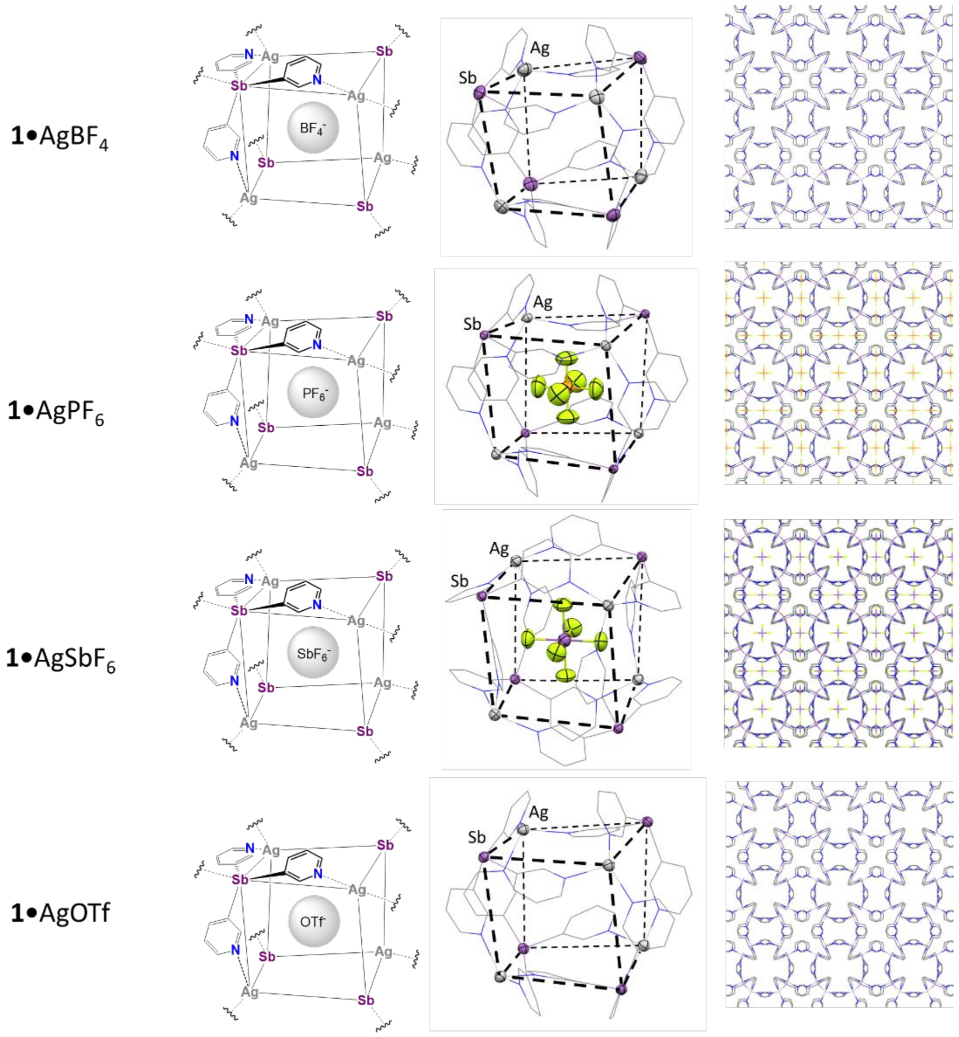

*Figure S17. Compounds 1•AgBF$_4$, 1•AgPF$_6$, 1•AgSbF$_6$ and 1•AgOTf: (left) Schematic representation of the formation of the monomeric cubane unit. (center) Crystal structure showing the heterocubane monomeric unit [Ag(**1**)(⊂X)]$^{3+}$ (X = BF$_4^-$, PF$_6^-$, SbF$_6^-$ or OTf) of the 3D-MOF. In the cases of 1•AgBF$_4$ and 1•AgOTf , disordered BF$_4^-$ and OTf$^-$ anions, respectively, are omitted for clarity. In the case of 1•AgOTf, the OTf$^-$ anions were not clearly resolved and therefore SQUEEZE was applied (see details above). Wireframe X-ray structure along the a, b or c axis of the 3D structure of the compound MOFs. Displacement ellipsoids at 50% probability. H atoms are omitted for clarity. Colour key: C (gray), Ag (light gray), P (light orange), Sb (light purple), N (blue) and F (yellow).*

2•CuPF$_6$

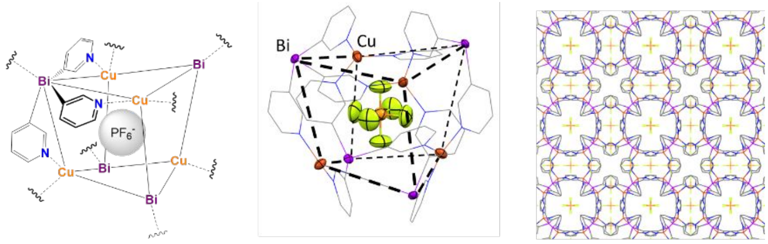

2•AgSbF$_6$

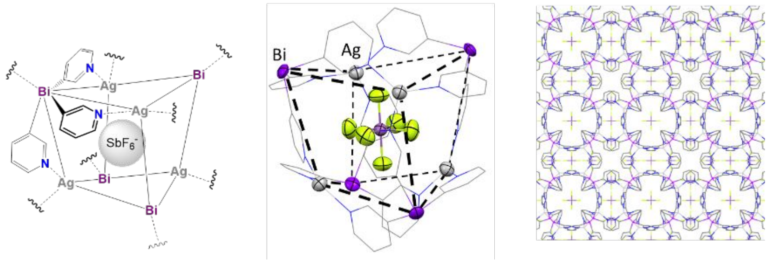

*Figure S18. Compounds 2•CuPF$_6$ and 2•AgSbF$_6$: (left) Schematic representation of the formation of the monomeric cubane unit. (center) Crystal structure showing the heterocubane monomeric unit [M(**1**)(⊂X)]$^{3+}$(M = Cu or Ag) (X = PF$_6^-$ or SbF$_6^-$) of the 3D-MOF. (right) Wireframe X-ray structure along the a, b or c axis of the 3D structure of the compound MOFs. Displacement ellipsoids at 50% probability. H atoms are omitted for clarity. Colour key: C (gray), Ag (light gray), Cu (orange), P (light orange), Sb (light purple), N (blue), Bi (purple) and F (yellow).*

Analysing the X-ray structures of the MOFs, we found that in those containing the Sb ligand, the pyramidalization of the metal atom (Cu or Ag) was much greater than that in those containing the Bi ligand. The N–M–Sb (M = Cu, Ag) angle varies between 108.3 and 112.6° (Fig. S19 left), while the N–M–Bi (M = Cu, Ag) angle takes the values 93.7 and 96.0° (Fig. S19 right). The rather unusual, almost planar coordination of the pyridyl-N atoms of the Bi-ligand to the Cu(I) and Ag(I) centers can be seen from the sums of the N–M–N bond angles (ΣMα, M = Cu, Ag), which are very close to 360 (Fig S20, right).

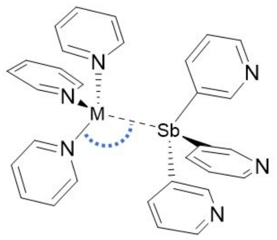

Compound	N-M-Sb
**1**•$CuBF_4$	108.3(2)
**1**•$CuPF_6$	108.9(2)
**1**•$AgBF_4$	111.4(2)
**1**•$AgPF_6$	112.3(2)
**1**•$AgSbF_6$	112.6(2)
**1**•AgOTf	111.6(2)

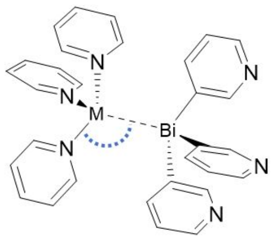

Compound	N-M-Bi
**2**•$CuPF_6$	96.0(2)
**2**•$AgSbF_6$	93.7(5)

Figure S19. (Left) Values for the angle N–M–Sb (M = Cu, Ag) for the Sb MOFs obtained from the corresponding X-ray structures. (Right) Values for the angle N–M–Bi (M = Cu, Ag) for the Bi MOFs obtained from the corresponding X-ray structures.

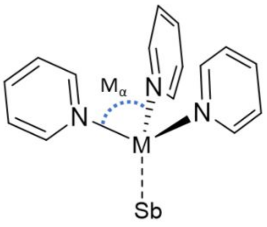

Compound	ΣM_α
**1**•$CuBF_4$	331.8
**1**•$CuPF_6$	330.0
**1**•$AgBF_4$	322.5
**1**•$AgPF_6$	319.5
**1**•$AgSbF_6$	318.6
**1**•AgOTf	321.9

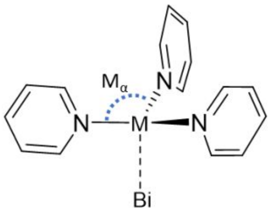

Compound	ΣM_α
**2**•$CuPF_6$	356.7
**2**•$AgSbF_6$	358.8

Figure S20. (Left) Values of the summation of the three angles N–M–N (ΣM_α) (M = Cu, Ag) for the Sb MOFs obtained from the corresponding X-ray structures. (Right) Values for the summation of the three angles N–M–N (ΣM_α) (M = Cu, Ag) for the Bi MOFs obtained from the corresponding X-ray structures.

Void analysis

Compound	Cage void volume [Å³]	Anion volume [Å³]
**1**•$CuBF_4$	125.5	53.4
**1**•$CuPF_6$	138.4	73.0
**1**•$AgBF_4$	102.8	53.4
**1**•$AgPF_6$	113.0	73.0
**1**•$AgSbF_6$	116.1	88.7
**1**•AgOTf	123.5	86.9

Table S1. Void volumes of the cage units for the Sb MOFs and anion volume for the corresponding anion (as determined using quantum-chemical calculations according to ref. 45 in the main manuscript).

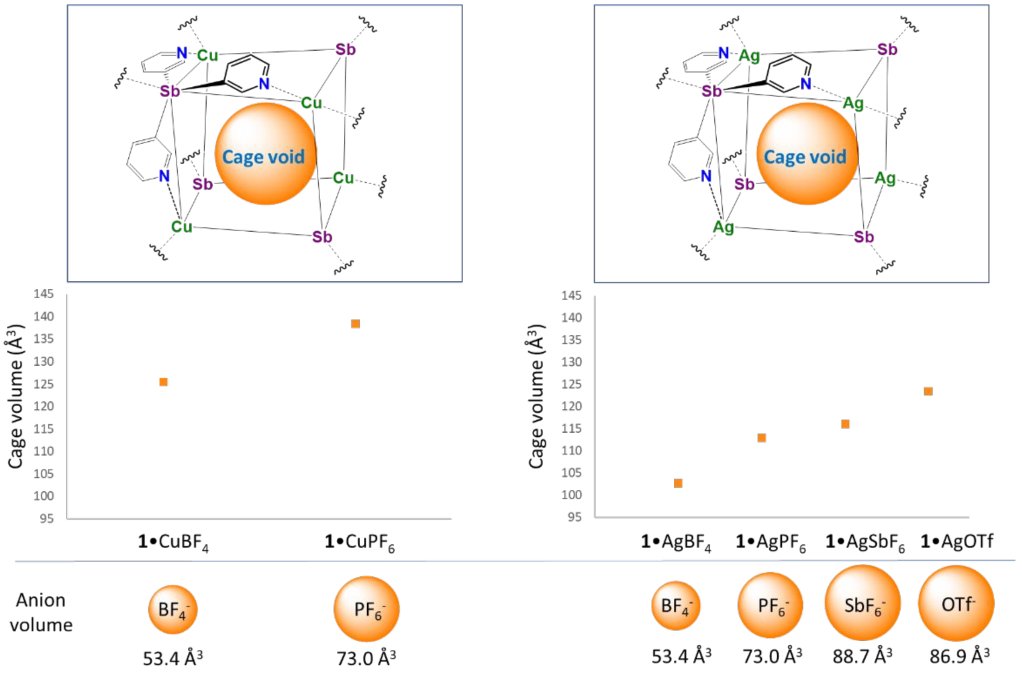

Figure S21. Representation of the void volumes of the cage units for the Sb MOFs depending on the encapsulated anion.

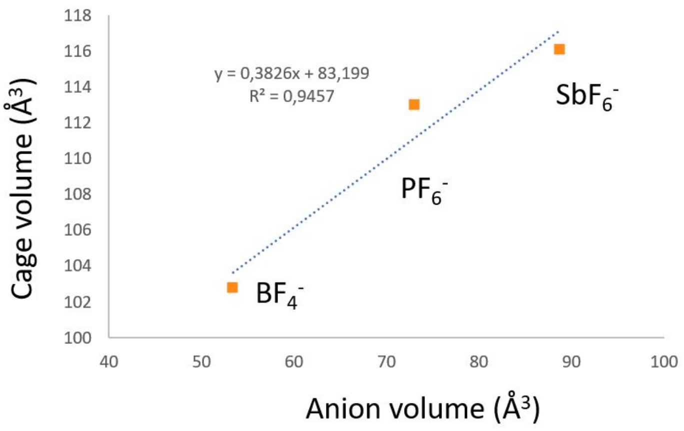

*Figure S22. Linear relationship observed between the cage volume and anion volume (ref. 45 in the main manuscript) for **1**•$AgBF_4$, **1**•$AgPF_6$ and **1**•$AgSbF_6$ (according to this correlation, **1**•AgOTf would have an anion volume closer to 105 Å^3).*

Table S2. Crystallographic data

Identification code	**1**	**2**	**1•**$CuBF_4$	**1•**$CuPF_6$	**1•**$AgBF_4$
CCDC Number	2110784	2110788	2110793	2110786	2110792
Empirical formula	$C_{15}H_{12}N_3Sb$	$C_{15}H_{12}BiN_3$	$C_{15}H_{12}BCuF_4N_3Sb$	$C_{15}H_{12}CuF_6N_3PSb$	$C_{15}H_{12}AgBF_4N_3Sb$
Formula weight	356.03	443.26	506.38	564.54	550.71
Temperature [K]	180(2)	293(2)	293(2)	293(2)	293(2)
Crystal system	monoclinic	monoclinic	cubic	cubic	cubic
Space group	$P2_1/c$	I2/a	P-43n	P-43n	P-43n
a [Å]	28.1372(11)	12.2816(5)	15.08330(11)	15.2117(2)	15.3710(2)
b [Å]	8.0071(3)	8.1825(4)	15.08330(11)	15.2117(2)	15.3710(2)
c [Å]	12.2521(5)	28.5136(14)	15.08330(11)	15.2117(2)	15.3710(2)
α [°]	90	90	90	90	90
β [°]	96.259(4)	98.764(4)	90	90	90
γ [°]	90	90	90	90	90
V [$Å^3$]	2743.91(18)	2832.0(2)	3431.54(8)	3519.92(14)	3631.69(14)
Z	8	8	8	8	8
ρcalc[g/cm^3]	1.724	2.079	1.960	2.131	2.014
μ [mm^{-1}]	2.000	12.440	2.856	2.900	2.606
F(000)	1392.0	1648.0	1952.0	2176.0	2096.0
Crystal size [mm^3]	0.30 × 0.23 × 0.07	0.34 × 0.22 × 0.08	0.19 × 0.16 × 0.09	0.18 × 0.16 × 0.08	0.23 × 0.19 × 0.11
Radiation	MoKα	MoKα	MoKα	MoKα	MoKα
2θ range for data collection [°]	6.69 to 52.758	6.604 to 59.108	6.616 to 58.864	6.56 to 53.484	6.492 to 59.166
Reflections collected	13954	11651	36007	23475	30433
Independent reflections	5586 [R_{int} = 0.0299, R_{sigma} = 0.0431]	3524 [R_{int} = 0.0397, R_{sigma} = 0.0394]	1586 [R_{int} = 0.0363, R_{sigma} = 0.0143]	1255 [R_{int} = 0.0402, R_{sigma} = 0.0143]	1671 [R_{int} = 0.0388, R_{sigma} = 0.0182]
Data/restraints /parameters	5586/0/343	3524/0/172	1586/60/133	1255/3/83	1671/60/133
GOF on F^2	1.009	1.035	1.112	1.063	1.032
Final R indexes [I>=2σ (I)]	R_1 = 0.0374, wR_2 = 0.0845	R_1 = 0.0280, wR_2 = 0.0494	R_1 = 0.0405, wR_2 = 0.1093	R_1 = 0.0383, wR_2 = 0.1197	R_1 = 0.0421, wR_2 = 0.1181
Final R indexes [all data]	R_1 = 0.0544, wR_2 = 0.0952	R_1 = 0.0391, wR_2 = 0.0546	R_1 = 0.0562, wR_2 = 0.1185	R_1 = 0.0471, wR_2 = 0.1297	R_1 = 0.0847, wR_2 = 0.1470
Max/min Δρ [eÅ-3]	2.32/-0.63	0.95/-1.25	0.45/-1.14	0.57/-1.40	0.32/-0.82
Flack parameter	-	-	0.005(12)	-0.027(16)	0.01(2)

Identification code	**1**•$AgPF_6$	**1**•$AgSbF_6$	**1**•AgOTf	**2**•$CuPF_6$	**2**•$AgSbF_6$
CCDC Number	2110782	2110787	2110783	2110791	2110785
Empirical formula	$C_{15}H_{12}AgF_6N_3PSb$	$C_{15}H_{12}AgF_6N_3Sb_2$	$AgC_{16}F_3H_{12}N_3O_3SSb$	$C_{15}H_{12}BiCuF_6N_3P$	$C_{15}H_{12}N_3F_6AgSbBi$
Formula weight	608.87	699.65	612.97	651.77	786.88
Temperature [K]	293(2)	293(2)	293(2)	220(2)	220(2)
Crystal system	cubic	cubic	cubic	cubic	cubic
Space group	P-43n	P-43n	P-43n	P-43n	P-43n
a [Å]	15.5531(3)	15.7019(3)	15.72180(15)	15.30475(19)	15.7606(6)
b [Å]	15.5531(3)	15.7019(3)	15.72180(15)	15.30475(19)	15.7606(6)
c [Å]	15.5531(3)	15.7019(3)	15.72180(15)	15.30475(19)	15.7606(6)
α [°]	90	90	90	90	90
β [°]	90	90	90	90	90
γ [°]	90	90	90	90	90
V [$Å^3$]	3762.3(2)	3871.3(2)	3886.03(11)	3584.92(13)	3914.9(4)
Z	8	8	8	8	8
ρcalc[g/cm^3]	2.150	2.401	2.095	2.415	2.670
μ [mm^{-1}]	2.623	3.833	2.556	11.144	11.388
F(000)	2320.0	2608.0	2352.0	2432.0	2864.0
Crystal size [mm^3]	0.29 × 0.22 × 0.12	0.37 × 0.26 × 0.24	0.34 × 0.27 × 0.22	0.20 × 0.16 × 0.08	0.12 × 0.10 × 0.08
Radiation	MoKα	MoKα	MoKα	MoKα	MoKα
2θ range for data collection [°]	7.41 to 59.384	7.34 to 58.422	7.332 to 59.504	6.52 to 59.09	7.312 to 51.332
Reflections collected	3730	4105	27599	25132	3273
Independent reflections	1443 [R_{int} = 0.0294, R_{sigma} = 0.0397]	1502 [R_{int} = 0.0296, R_{sigma} = 0.0368]	1753 [R_{int} = 0.0347, R_{sigma} = 0.0156]	1641 [R_{int} = 0.0445, R_{sigma} = 0.0228]	1181 [R_{int} = 0.0581, R_{sigma} = 0.0711]
Data/restraints/parameters	1443/0/83	1502/0/83	1753/0/61	1641/0/83	1181/0/83
GOF on F^2	1.043	1.076	1.067	1.064	1.064
Final R indexes [I>=2σ (I)]	R_1 = 0.0375, wR_2 = 0.0835	R_1 = 0.0344, wR_2 = 0.0729	R_1 = 0.0436, wR_2 = 0.1092	R_1 = 0.0291, wR_2 = 0.0677	R_1 = 0.0497, wR_2 = 0.0951
Final R indexes [all data]	R_1 = 0.0657, wR_2 = 0.1009	R_1 = 0.0529, wR_2 = 0.0822	R_1 = 0.0633, wR_2 = 0.1200	R_1 = 0.0512, wR_2 = 0.0764	R_1 = 0.0992, wR_2 = 0.1195
Max/min Δρ [eÅ-3]	0.34/-0.93	0.57/-1.51	1.10/-1.01	0.64/-1.12	0.66/-1.95
Flack parameter	-0.10(5)	-0.01(4)	0.00(2)	-0.006(7)	-0.032(12)

Identification code	**2**•$CuBF_4$	**2**•LiBr•MeCN
CCDC Number	2110789	2110790
Empirical formula	$C_{30}H_{24}BBi_2CuF_4N_6$	$C_{17}H_{15}BiBrLiN_4$
Formula weight	1036.86	571.16
Temperature [K]	180(2)	180(2)
Crystal system	triclinic	triclinic
Space group	P-1	P-1
a [Å]	9.3481(9)	9.2995(4)
b [Å]	13.8529(13)	9.6180(4)
c [Å]	14.0214(13)	11.2939(5)
α [°]	113.223(4)	99.809(2)
β [°]	105.177(3)	97.8330(10)
γ [°]	99.420(4)	108.493(2)
V [$Å^3$]	1535.8(3)	924.20(7)
Z	2	2
ρcalc[g/cm^3]	2.242	2.052
μ [mm^{-1}]	12.172	11.704
F(000)	964.0	532.0
Crystal size [mm^3]	0.15 × 0.03 × 0.02	0.1 × 0.1 × 0.08
Radiation	MoKα	MoKα
2θ range for data collection [°]	4.728 to 48.944	6.448 to 55.028
Reflections collected	29918	28433
Independent reflections	5064 [R_{int} = 0.0673, R_{sigma} = 0.0439]	4181 [R_{int} = 0.0636, R_{sigma} = 0.0377]
Data/restraints/parameters	5064/0/397	4181/0/219
GOF on F^2	1.069	1.049
Final R indexes [I>=2σ (I)]	R_1 = 0.0392, wR_2 = 0.0873	R_1 = 0.0200, wR_2 = 0.0463
Final R indexes [all data]	R_1 = 0.0539, wR_2 = 0.0960	R_1 = 0.0229, wR_2 = 0.0472
Max/min Δρ [eÅ-3]	4.14/-2.13	0.99/-1.06
Flack parameter	-	-

Table S3. Selected bond length and angles.

(E=Sb,Bi; M=Cu,Ag,Li)	1	2	1•$CuBF_4$	1•$CuPF_6$	1•$AgBF_4$	1•$AgPF_6$
C_{py}-E	2.149(4)-2.160(4)	2.249(4)-2.261(4)	2.137(7)	2.132(8)	2.11(1)	2.146(8)
C_{py}-E-C_{py}	91.3(1)-98.5(2)	90.5(1)-96.3(1)	98.4(3)	98.7(3)	100.2(4)	100.4(3)
M---E	-	-	2.575(1)	2.596(1)	2.654(1)	2.6613(9)
N-M	-	-	2.052(6)	2.051(8)	2.281(8)	2.287(8)
N_{py}-M-N_{py}	-	-	110.6(3)	110.0(3)	107.5(3)	106.5(3)

(E=Sb,Bi; M=Cu,Ag,Li)	1•$AgSbF_6$	1•AgOTf	2•$CuPF_6$	2•$AgSbF_6$	2•$CuBF_4$	2•LiBr•MeCN
C_{py}-E	2.126(8)	2.115(8)	2.239(8)	2.26(2)	2.26(1)-2.29(1)	2.256(4)-2.286(3)
C_{py}-E-C_{py}	100.4(3)	100.8(3)	94.3(3)	93.9(8)	91.1(5)-95.3(5)	91.8(1)-92.9(1)
M---E	2.6884(9)	2.7092(9)	3.041(2)	3.178(2)	-	-
N-M	2.305(8)	2.310(8)	1.987(6)	2.21(2)	2.03(1)-2.07(1)	2.050(7)-2.075(5)
N_{py}-M-N_{py}	106.2(3)	107.3(3)	118.9(3)	119.6(7)	105.7(4)-114.9(4)	104.3(3)-116.3(3)

Powder X-ray studies

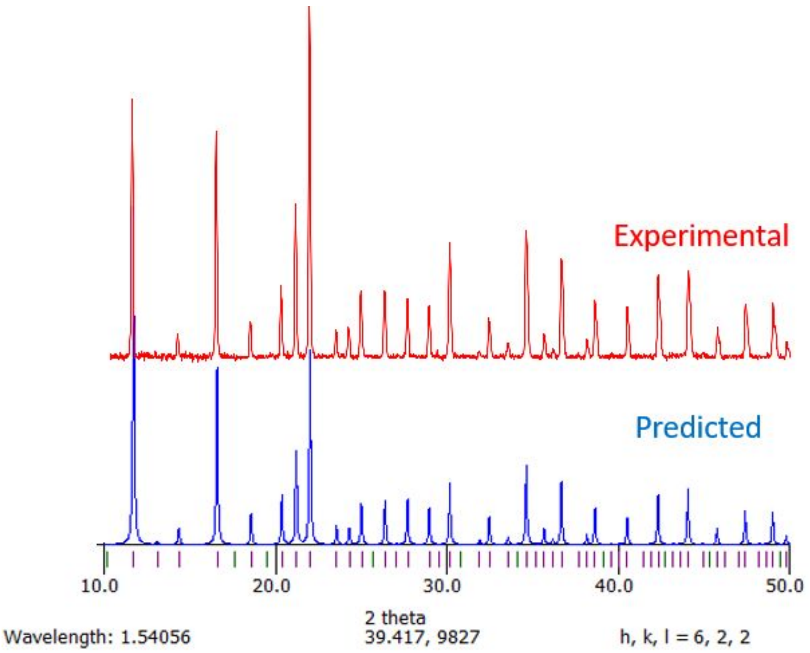

*Figure S23. Comparison of the experimental (above, red) and the predicted (below, blue) XRPD patterns for compound **1**•$CuBF_4$.*

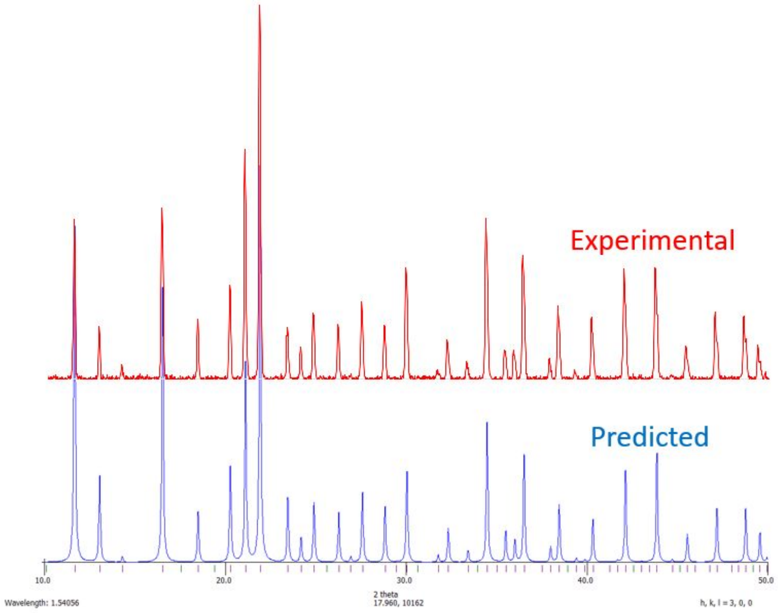

*Figure S24. Comparison of the experimental (above, red) and the predicted (below, blue) XRPD patterns for compound **1**•$CuPF_6$.*

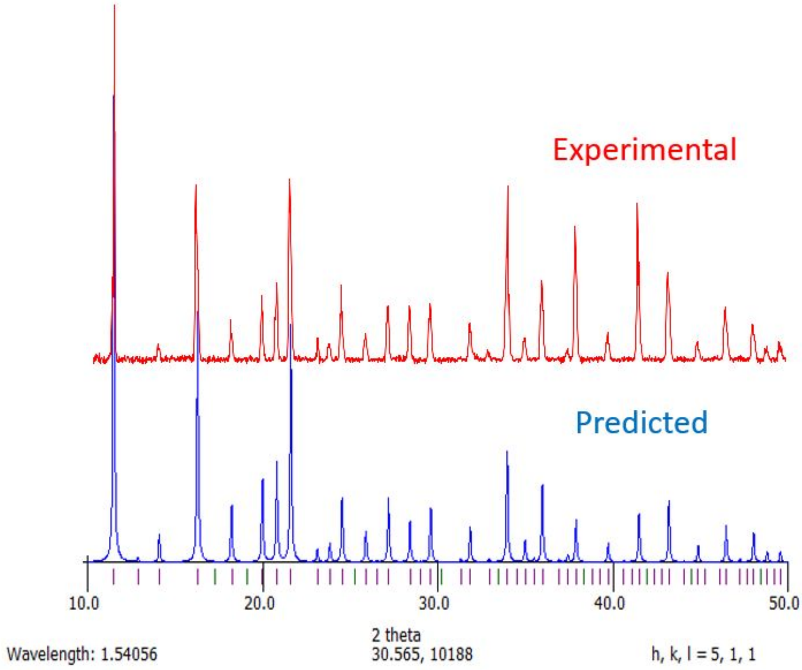

*Figure S25. Comparison of the experimental (above, red) and the predicted (below, blue) XRPD patterns for compound **1**•$AgBF_4$.*

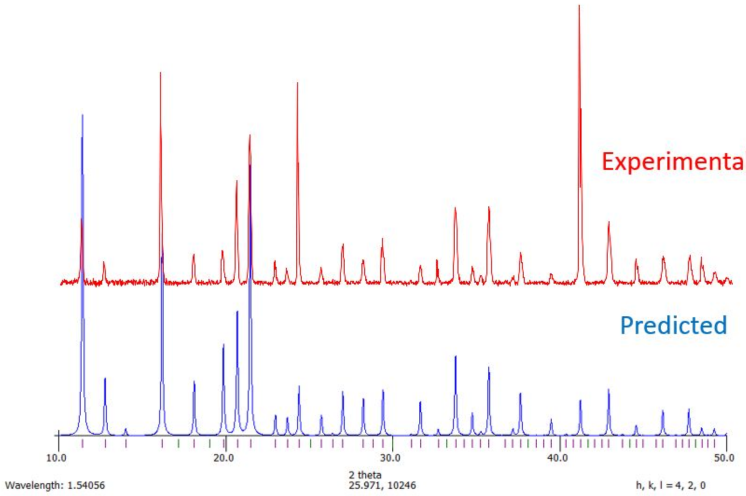

*Figure S26. Comparison of the experimental (above, red) and the predicted (below, blue) XRPD patterns for compound **1**•$AgPF_6$.*

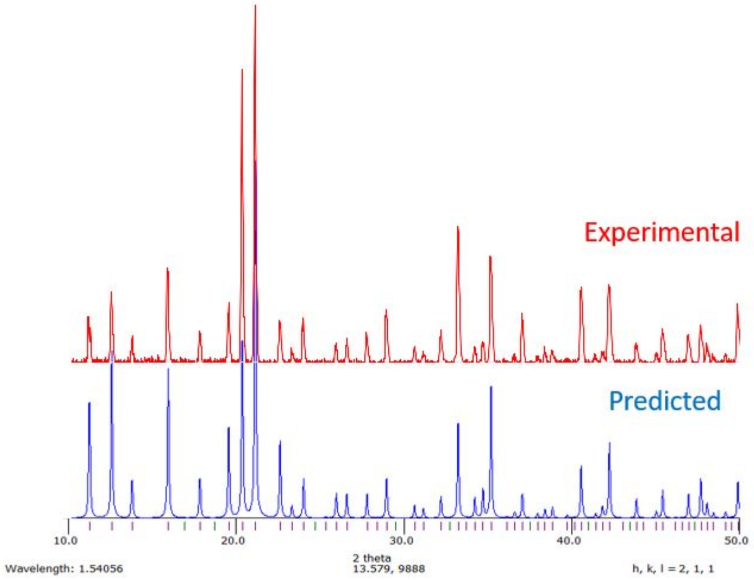

*Figure S27. Comparison of the experimental (above, red) and the predicted (below, blue) XRPD patterns for compound **1**•$AgSbF_6$.*

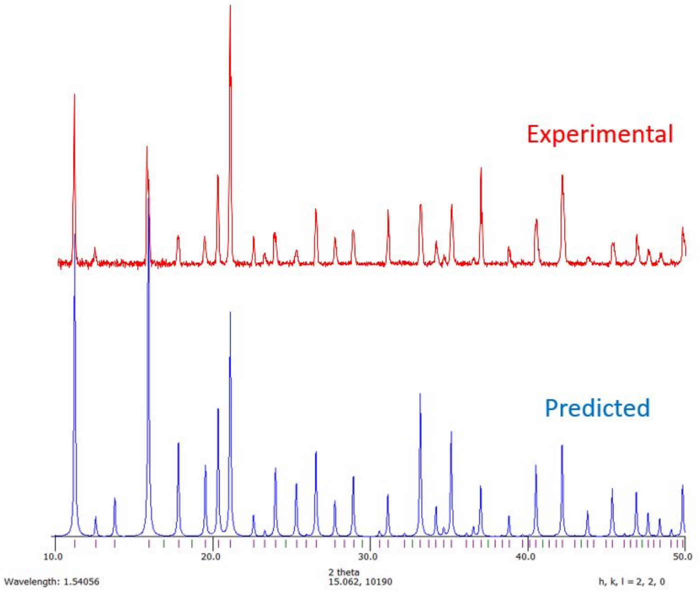

*Figure S28. Comparison of the experimental (above, red) and the predicted (below, blue) XRPD patterns for compound **1**•AgOTf.*

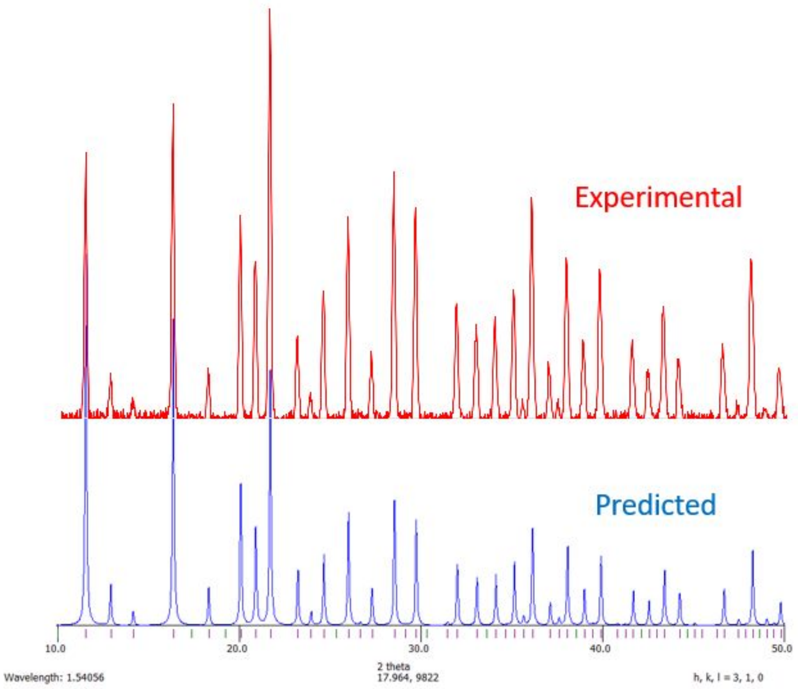

*Figure S29. Comparison of the experimental (above, red) and the predicted (below, blue) XRPD patterns for compound **2**•$CuPF_6$.*

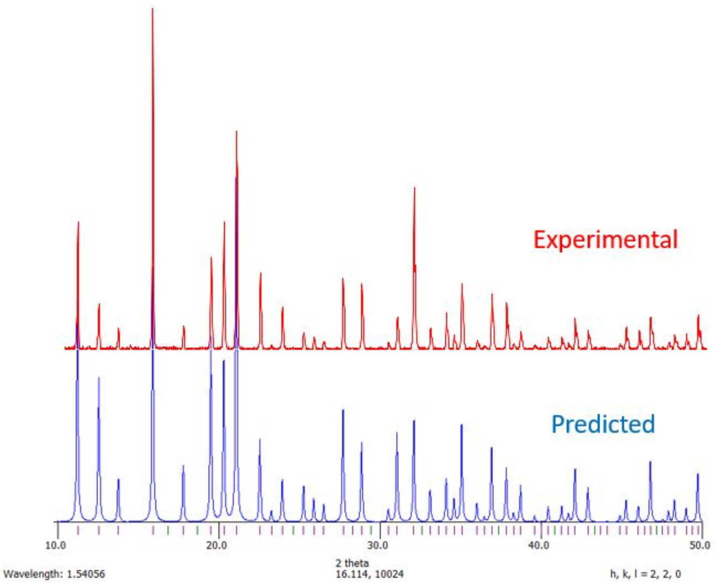

*Figure S30. Comparison of the experimental (above, red) and the predicted (below, blue) XRPD patterns for compound **2**•$AgSbF_6$.*

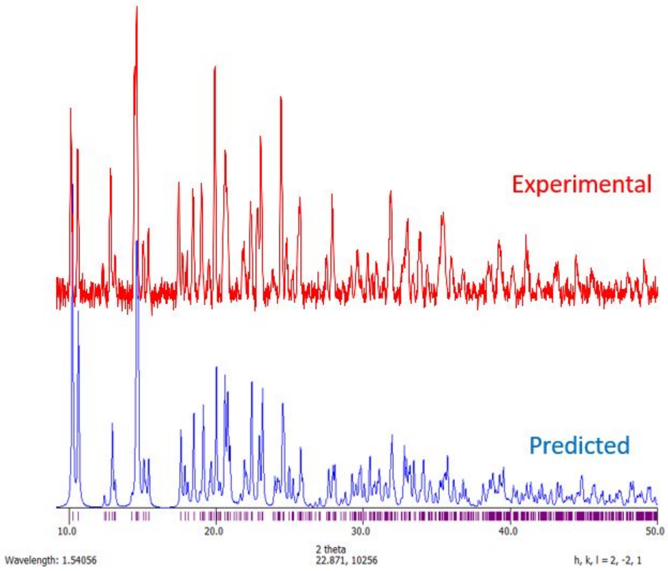

*Figure S31. Comparison of the experimental (above, red) and the predicted (below, blue) XRPD patterns for compound **2**•$CuBF_4$.*

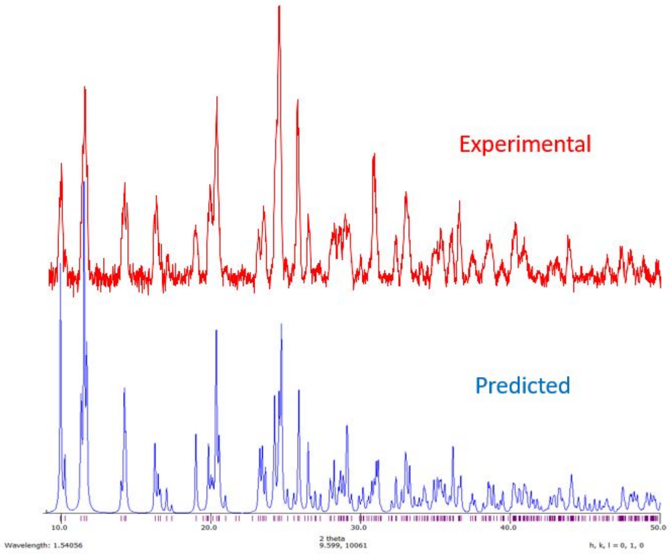

*Figure S32. Comparison of the experimental (above, red) and the predicted (below, blue) XRPD patterns for compound **2**•LiBr•MeCN.*

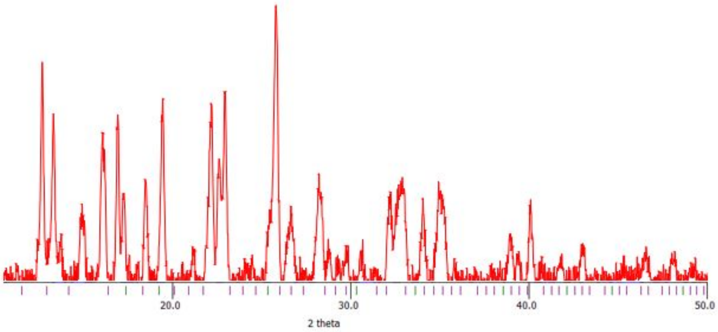

*Figure S33. Experimental XRPD pattern for the yellow insoluble solid obtained from the reaction of equimolar amounts of **2** and CuBr, indicating a different outcome from the 3D-MOFs (see XRPD patterns for 3D MOFs shown previously).*

Emission studies for selected copper complexes

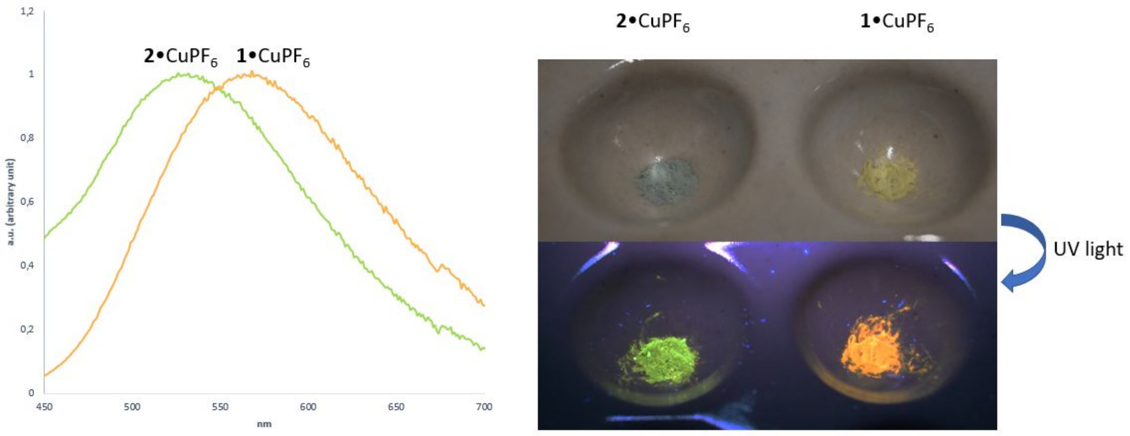

*Figure S34. (Left) Emission spectra (room temperature) of antimony MOF **1**•CuPF$_6$ and bismuth MOF **2**•CuPF$_6$ as crystal powders at room temperature. The samples were excited at λ_{exc} = 375 nm. (Right) Pictures of **1**•CuPF$_6$ and **2**•CuPF$_6$ after grinding the samples under natural light (above) and under UV lamp light at 365 nm (below). Note: In both cases, no changes were observed in the emission upon grinding the samples.*

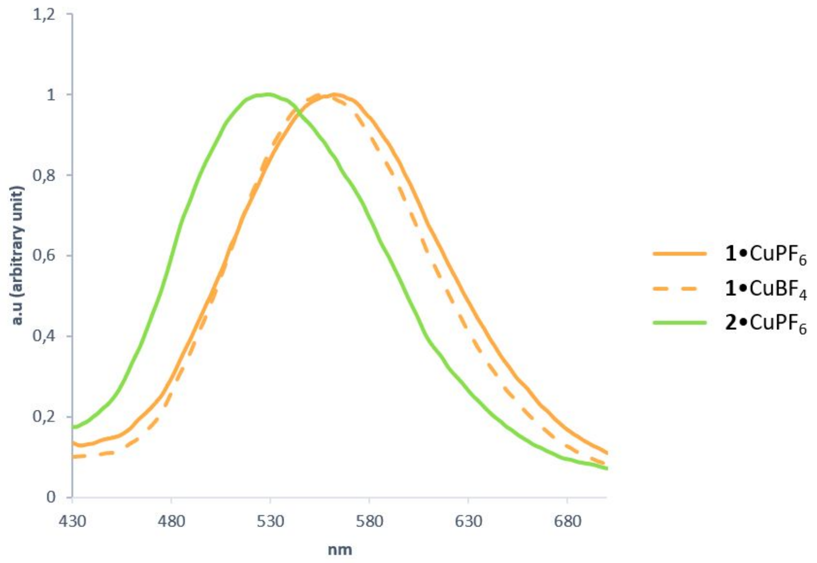

*Figure S35. Emission spectra (room temperature, crystal powders) of antimony **1**•CuPF$_6$ and **1**•CuBF$_4$ MOFs and bismuth MOF **2**•CuPF$_6$. While changing the anion does not substantially change the emission spectra (cf. **1**•CuPF$_6$ and **1**•CuBF$_4$), changing the bridgehead has a larger impact on the luminescence properties of the MOF (cf. **1**•CuX (X = BF_4^-, PF_6^-) and **2**•CuPF$_6$).*

Compound	λ_{exc}	λ_{emis}	Φ (%)	$\tau_{av}{}^{a}$(ns)	$\tau_n{}^{b}$; $A_n{}^{c}$
**1**•$CuPF_6$	**375**	**570**	**8.0**	**7.58**	**τ_1=1.63; A_1=0.45**<br>**τ_2=8.52; A_2=0.55**
**2**•$CuPF_6$	**375**	**530**	**0.31**	**6.16**	**τ_1=1.50; A_1=0.33**<br>**τ_2=6.68; A_2=0.67**

*Table S4. Excitation and emission data (nm) (solid state, room temperature) for crystal powders of **1**•$CuPF_6$ and **2**•$CuPF_6$. Average lifetime ^{a} $\tau_{av} = (A_1\tau_1^2 + A_2\tau_2^2) / (A_1\tau_1 + A_2\tau_2)$. ^{b} τ_n = Natural lifetime. ^{c} A_n = Intensity coefficient.*

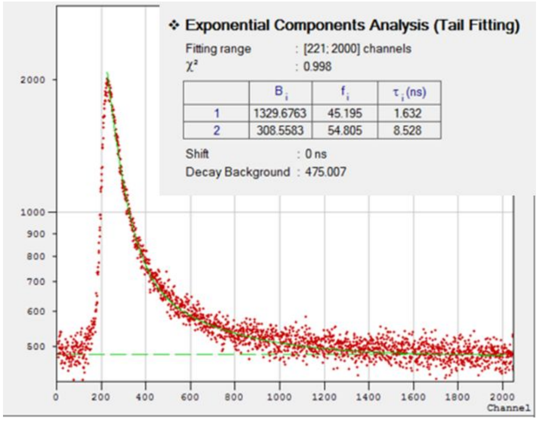

*Figure S36. Exponential components analysis for **1**•$CuPF_6$.*

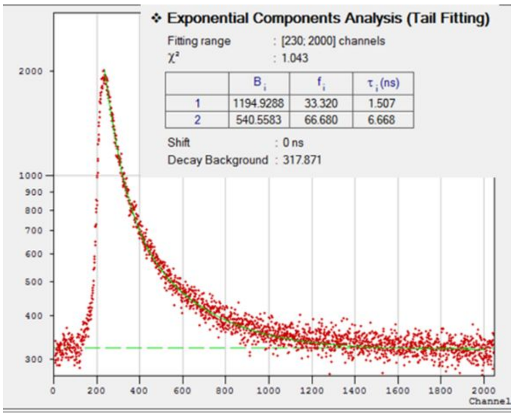

*Figure S37. Exponential components analysis for **2**•$CuPF_6$.*

Computational details

All computations were carried out using the Gaussian16 package,[2] in which the hybrid method of Austin, Petersson and Frisch with spherical atom dispersion terms (APFD) was applied.[3] The triple zeta cc-pVTZ-PP basis set with effective core potentials was used for the heavy atoms (Sb, Bi, Cu, Ag),[4–7] as found in the basis set exchange library,[8] and 6-31G(d') was used for the rest of the atoms. Geometry optimizations were performed without symmetry restrictions using the initial coordinates derived from X-ray data when available, and frequency analyses were performed to ensure that a minimum structure with no imaginary frequencies was achieved in each case. Energy Decomposition Analysis (EDA) was performed on the geometry optimized models and on the X-ray geometry models with the AOMix program.[9,10] NBO analysis was performed with the NBO 7.0 program.[11] The visualization of the calculation results was performed with GaussView 6.1.[12]

Optimization of the model structure

The X-ray derived model structure of the compound 2•$AgSbF_6$ was optimized without symmetry restrictions using various functionals, and, in all cases, the Bi-Ag distance was found to be shorter than in the X-ray structure.

Functional	Bi-Ag distance (Å)
APFD	2.655
B3LYP	2.869
M06	2.962
TPSSh	2.794
**X-ray**	3.180

Table S5. Bi-Ag distance (for compound 2•$AgSbF_6$) for various functionals.

Study of the E → M interaction

The energy of the E-M interaction was calculated using an Energy Decomposition Analysis (EDA) that was carried out on both the optimized structures and the X-ray derived structures. The composition of the orbitals involved in the interaction was studied using a NBO analysis.

Compound	M-E Distance (Å)	Sum of angles N-M-N	Energy E-M (kcal/mol)	Composition Lp(E) → Lv(M)
1•$CuPF_6$ (xray)	2.596	330.0	-34.2	69% s + 31% p → 100% s
1•$CuPF_6$ (opt)	2.399	324.6	-39.3	67% s + 33% p → 100% s
2•$CuPF_6$ (xray)	3.041	356.7	-15.0	79% s + 21% p → 98% s
2•$CuPF_6$ (opt)	2.501	339.3	-29.0	79% s + 21% p → 99% s
1•$AgSbF_6$ (xray)	2.661	318.6	-35.2	66% s + 34% p → 100% s
1•$AgSbF_6$ (opt)	2.517	305.1	-39.5	67% s + 33% p → 100% s
2•$AgSbF_6$ (xray)	3.178	358.8	-14.1	80% s + 20% p → 98% s
2•$AgSbF_6$ (opt)	2.655	341.4	-27.0	80% s + 20% p → 98% s

Table S6. Energy of the E-M interaction and composition of the orbital involved

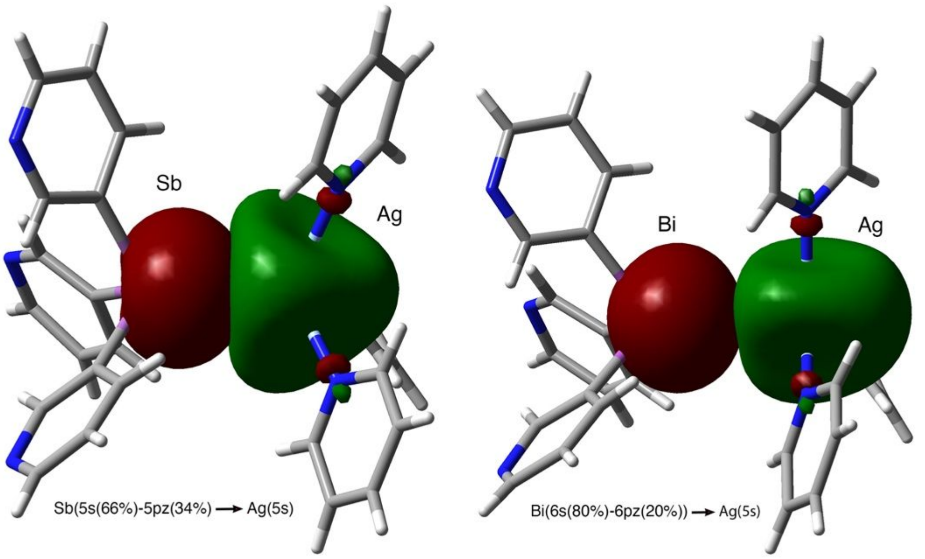

Figure S38. Simple model of the Bi MOF 2•$AgSbF_6$ by taking a fragment of the extended X-ray structure consisting of a $[Ag(py)_3]^+$ moiety attached to a $Bi(3\text{-}py)_3$ ligand.

1•$CuPF_6$ **optimized structure coordinates**

C	2.99164500	-2.18789700	-3.52081800
N	0.69278900	1.73450300	-2.15378600
C	0.39895200	3.68478900	-3.52081800
C	0.00000000	2.43218400	-3.06660200
H	-0.90711800	1.95643600	-3.43348000
C	1.82148600	2.27305800	-1.66618500
H	2.34592000	1.67897300	-0.92109100
C	1.57069400	4.24043100	-3.00785600
H	1.91102800	5.21929600	-3.33947900
C	2.29707400	3.51835100	-2.06208500
H	3.21497100	3.90952800	-1.63071000
Cu	0.00000000	0.00000000	-1.44716500
N	-1.84851800	-0.26727900	-2.15378600
C	-3.39059700	-1.49689200	-3.52081800
C	-2.10633300	-1.21609200	-3.06660200
H	-1.24076500	-1.76380500	-3.43348000
C	-2.87926800	0.44092400	-1.66618500
H	-2.62699300	1.19214000	-0.92109100
C	-4.45766800	-0.75995500	-3.00785600
H	-5.47555700	-0.95464900	-3.33947900
C	-4.19551800	0.23014900	-2.06208500
H	-4.99323600	0.82948200	-1.63071000
Sb	0.00000000	0.00000000	0.95150600
N	1.15572900	-1.46722500	-2.15378600
C	2.10633300	-1.21609200	-3.06660200
H	2.14788200	-0.19263100	-3.43348000
C	1.05778300	-2.71398200	-1.66618500
H	0.28107300	-2.87111200	-0.92109100
C	2.88697400	-3.48047700	-3.00785600
H	3.56452900	-4.26464700	-3.33947900
C	1.89844400	-3.74849900	-2.06208500
H	1.77826500	-4.73901000	-1.63071000
N	2.30271400	2.54575700	3.68426700
C	0.86914700	1.67206300	1.94874900
C	1.71349300	1.52616700	3.05781200
H	1.92865000	0.53660800	3.46371100
C	2.06103600	3.77366100	3.22248000
H	2.55322400	4.58663500	3.75726300
C	0.63595000	2.97040700	1.47606700
H	-0.00838400	3.14522900	0.61357100
C	1.23696300	4.04368800	2.12732500
H	1.07364100	5.06770600	1.79795500
N	-3.35604700	0.72133000	3.68426700
C	-1.88262300	-0.08332900	1.94874900
C	-2.17844600	0.72084500	3.05781200
H	-1.42904100	1.40195500	3.46371100
C	-4.29860500	-0.10192100	3.22248000
H	-5.24875500	-0.08216100	3.75726300
C	-2.89042300	-0.93445500	1.47606700
H	-2.71965600	-1.57987600	0.61357100

C	-4.12041800	-0.95060300	2.12732500
H	-4.92558300	-1.60405300	1.79795500
C	1.01347600	-1.58873500	1.94874900
N	1.05333400	-3.26708700	3.68426700
C	0.46495300	-2.24701200	3.05781200
H	-0.49960800	-1.93856400	3.46371100
C	2.23756800	-3.67174000	3.22248000
H	2.69553000	-4.50447500	3.75726300
C	2.25447300	-2.03595200	1.47606700
H	2.72804100	-1.56535400	0.61357100
C	2.88345500	-3.09308500	2.12732500
H	3.85194200	-3.46365400	1.79795500
H	-3.54583100	-2.27808400	-4.26077200
H	-0.19996300	4.20982200	-4.26077200
H	3.74579400	-1.93173800	-4.26077200

2•$CuPF_6$ **optimized structure coordinates**

C	-3.40837200	-3.43613600	1.80523700
Cu	-1.74064700	-0.00281400	-0.00341000
N	-2.26424300	0.59888200	-1.81843800
C	-3.36605500	0.13957200	-3.89983100
C	-1.73580800	1.70452100	-2.36676400
H	-1.08359700	2.28907400	-1.72163700
C	-3.06777700	-0.16426500	-2.57619000
H	-3.47441600	-1.04826200	-2.08924100
C	-1.97762100	2.08524800	-3.68191400
H	-1.51654300	2.98652700	-4.07751200
C	-2.80833000	1.28605100	-4.46570800
H	-3.01836300	1.55136200	-5.49983000
N	-2.28032700	1.26896500	1.41866800
C	-3.41040800	3.28929300	2.05011200
C	-1.75488500	1.20265500	2.65242800
H	-1.09246300	0.36093800	2.84205200
C	-3.09612800	2.29532700	1.12998800
H	-3.49949500	2.30643400	0.11950200
C	-2.01203000	2.15209700	3.63500800
H	-1.55276500	2.05396000	4.61523900
C	-2.85572900	3.21836900	3.32793800
H	-3.07790500	3.98146500	4.07105900
Bi	0.76055100	-0.00234100	0.00418700
N	-2.27600200	-1.87229300	0.38063500
C	-1.74314500	-2.90433600	-0.29273000
H	-1.07647400	-2.64338800	-1.11171700
C	-3.09644500	-2.14040300	1.40888100
H	-3.50528700	-1.27337900	1.92367400
C	-1.99783500	-4.23159300	0.03372700
H	-1.53270000	-5.02859400	-0.54059800
C	-2.84653300	-4.50395900	1.10548700
H	-3.06698200	-5.53041000	1.39132600
N	3.53471600	2.45106200	-2.51642300

C	1.85399000	0.92005800	-1.70836200
C	3.15813600	2.15713800	-3.76190800
H	3.70273700	2.67020700	-4.55529200
C	2.89392300	1.83799200	-1.51897600
H	3.23149900	2.09424700	-0.51359800
C	2.13793700	1.25551500	-4.07303700
H	1.87900200	1.05008300	-5.10982700
C	1.46977700	0.63047400	-3.02263200
H	0.66587700	-0.07634400	-3.23281200
N	3.55176600	-3.40312200	-0.84054500
C	1.85728500	-1.94471000	0.06688200
C	3.16823000	-4.33806800	0.03024900
H	3.71572400	-5.28017700	-0.01404500
C	2.90758600	-2.23457200	-0.81231500
H	3.25108500	-1.48953200	-1.53158900
C	2.13769000	-4.16172800	0.95619600
H	1.87338600	-4.95938900	1.64762200
C	1.46624500	-2.94126600	0.96837500
H	0.65436000	-2.77368300	1.67746700
C	1.84869600	1.02621500	1.65875300
N	3.52981900	0.97238000	3.38866700
C	3.14660900	2.19644000	3.75493000
H	3.68886700	2.63115500	4.59532200
C	2.89200400	0.40993400	2.36002900
H	3.23548700	-0.58735000	2.08097800
C	2.12253500	2.91026200	3.12865100
H	1.85823100	3.90997600	3.46762200
C	1.45756100	2.30778700	2.06309000
H	0.65079500	2.83792000	1.55499200
H	-4.02132200	-0.51272900	-4.47160800
H	-4.07533200	4.10096700	1.76574800
H	-4.07710700	-3.59976400	2.64655400

1•$AgSbF_6$ **optimized structure coordinates**

N	1.97511100	-0.37512800	-2.28006000
C	3.64144700	0.26208300	-3.88362200
C	2.38068000	0.38356600	-3.30673600
H	1.65919300	1.11513900	-3.66663200
C	2.82415000	-1.28788500	-1.78736200
H	2.45729000	-1.87397100	-0.94706000
C	4.10426300	-1.48041200	-2.29781400
H	4.75599000	-2.23346500	-1.86175100
C	4.52066900	-0.68957400	-3.36814300
H	5.51504500	-0.81177200	-3.79297400
Ag	0.00000000	0.00000000	-1.28207200
C	-2.04769400	3.02254400	-3.88362200
N	-1.31242600	-1.52293200	-2.28006000
C	-1.59375200	-3.28462700	-3.88362200
C	-0.85816200	-2.25351200	-3.30673600
H	0.13614300	-1.99447200	-3.66663200
C	-2.52741600	-1.80184300	-1.78736200

H	-2.85155200	-1.19109000	-0.94706000
C	-3.33420500	-2.81419000	-2.29781400
H	-4.31223300	-3.00207600	-1.86175100
C	-2.85752300	-3.57022700	-3.36814300
H	-3.46053800	-4.37028300	-3.79297400
Sb	0.00000000	0.00000000	1.23467200
N	-0.66268500	1.89806000	-2.28006000
C	-1.52251800	1.86994600	-3.30673600
H	-1.79533500	0.87933300	-3.66663200
C	-0.29673400	3.08972800	-1.78736200
H	0.39426200	3.06506100	-0.94706000
C	-0.77005700	4.29460200	-2.29781400
H	-0.44375700	5.23554100	-1.86175100
C	-1.66314600	4.25980100	-3.36814300
H	-2.05450700	5.18205500	-3.79297400
N	0.00000000	3.36662900	4.04134500
C	-0.42152200	1.83925300	2.22095700
C	0.22884900	2.21823200	3.40308700
H	0.97493600	1.56599400	3.85977500
C	-0.90548200	4.19282800	3.51514900
H	-1.07315200	5.12166800	4.06125100
C	-1.61441100	3.92414700	2.34181900
H	-2.34313500	4.63688100	1.96138100
C	-1.36270000	2.72519500	1.68103500
H	-1.89379200	2.48402800	0.75918100
N	2.91558600	-1.68331500	4.04134500
C	1.80360100	-0.55457700	2.22095700
C	1.80662100	-1.30730500	3.40308700
H	0.86872300	-1.62731600	3.85977500
C	4.08383600	-1.31224400	3.51514900
H	4.97207100	-1.63145700	4.06125100
C	4.20561600	-0.56395300	2.34181900
H	5.18722400	-0.28922600	1.96138100
C	3.04143900	-0.18246400	1.68103500
H	3.09812800	0.39805800	0.75918100
C	-1.38207800	-1.28467500	2.22095700
N	-2.91558600	-1.68331500	4.04134500
C	-2.03547000	-0.91092700	3.40308700
H	-1.84365900	0.06132200	3.85977500
C	-3.17835500	-2.88058400	3.51514900
H	-3.89891900	-3.49021100	4.06125100
C	-2.59120500	-3.36019400	2.34181900
H	-2.84408900	-4.34765500	1.96138100
C	-1.67873800	-2.54273100	1.68103500
H	-1.20433600	-2.88208600	0.75918100
H	-2.74301900	2.94731100	-4.71602100
H	-1.18093700	-3.84918000	-4.71602100
H	3.92395600	0.90186900	-4.71602100

2•AgSbF_6 **optimized structure coordinates**

N	0.57126500	2.07063600	-2.32792700
C	0.00000000	4.15079700	-3.37778800
C	-0.18903400	2.78853900	-3.16703000
H	-0.97687400	2.24005300	-3.68001400
C	1.81608900	4.05647600	-1.81063200
H	2.62195300	4.51614600	-1.24417600
C	1.02238300	4.79804400	-2.68424600
H	1.19682100	5.86316500	-2.82293000
C	1.55435400	2.69780500	-1.66639500
H	2.14092400	2.08267400	-0.98729400
Ag	0.00000000	0.00000000	-1.76583500
C	3.59469500	-2.07539800	-3.37778800
N	-2.07885600	-0.54058800	-2.32792700
C	-3.59469500	-2.07539800	-3.37778800
C	-2.32042900	-1.55797800	-3.16703000
H	-1.45150600	-1.96602400	-3.68001400
C	-4.42105500	-0.45545900	-1.81063200
H	-5.22207400	0.01260500	-1.24417600
C	-4.66641900	-1.51361200	-2.68424600
H	-5.67606100	-1.89510500	-2.82293000
C	-3.11354500	-0.00279300	-1.66639500
H	-2.87411100	0.81275800	-0.98729400
Bi	0.00000000	0.00000000	0.88889800
N	1.50759100	-1.53004800	-2.32792700
C	2.50946300	-1.23056200	-3.16703000
H	2.42837900	-0.27402900	-3.68001400
C	2.60496700	-3.60101700	-1.81063200
H	2.60012100	-4.52875100	-1.24417600
C	3.64403600	-3.28443200	-2.68424600
H	4.47923900	-3.96806000	-2.82293000
C	1.55919100	-2.69501300	-1.66639500
H	0.73318700	-2.89543200	-0.98729400
N	2.22839500	2.70205200	3.67956000
C	0.78865000	1.77933400	1.97841800
C	1.69611500	1.65993200	3.03779900
H	2.01255800	0.67714400	3.39046100
C	0.96080500	4.16296100	2.24323000
H	0.69247000	5.18132000	1.96912300
C	0.41961900	3.06734600	1.57446400
H	-0.28230400	3.22191300	0.75392200
C	1.86037100	3.92166300	3.28401300
H	2.30898200	4.75231200	3.82996100
N	-3.45424300	0.57882000	3.67956000
C	-1.93527400	-0.20667600	1.97841800
C	-2.28560100	0.63891300	3.03779900
H	-1.59270300	1.40435500	3.39046100
C	-4.08563200	-1.24939900	2.24323000
H	-4.83339000	-1.99096400	1.96912300
C	-2.86620900	-1.17027200	1.57446400
H	-2.64910600	-1.85543900	0.75392200

C	-4.32644500	-0.34970300	3.28401300
H	-5.27011400	-0.37651900	3.82996100
C	1.14662400	-1.57265800	1.97841800
N	1.22584900	-3.28087200	3.67956000
C	0.58948600	-2.29884500	3.03779900
H	-0.41985600	-2.08149800	3.39046100
C	3.12482800	-2.91356200	2.24323000
H	4.14092000	-3.19035600	1.96912300
C	2.44659000	-1.89707300	1.57446400
H	2.93141100	-1.36647400	0.75392200
C	2.46607400	-3.57196000	3.28401300
H	2.96113200	-4.37579400	3.82996100
H	4.38209200	-1.78677000	-4.06957700
H	-0.64365700	4.68838800	-4.06957700
H	-3.73843500	-2.90161800	-4.06957700

References

(1) A. L. Spek. PLATON SQUEEZE: A Tool for the Calculation of the Disordered Solvent Contribution to the Calculated Structure Factors. *Acta Crystallogr. C Struc. Chem.*, **2015**, *71*, 9–18.

(2) Gaussian 16, Revision C.01, M. J. Frisch, G. W. Trucks, H. B. Schlegel, G. E. Scuseria, M. A. Robb, J. R. Cheeseman, G. Scalmani, V. Barone, G. A. Petersson, H. Nakatsuji, X. Li, M. Caricato, A. V. Marenich, J. Bloino, B. G. Janesko, R. Gomperts, B. Mennucci, H. P. Hratchian, J. V. Ortiz, A. F. Izmaylov, J. L. Sonnenberg, D. Williams-Young, F. Ding, F. Lipparini, F. Egidi, J. Goings, B. Peng, A. Petrone, T. Henderson, D. Ranasinghe, V. G. Zakrzewski, J. Gao, N. Rega, G. Zheng, W. Liang, M. Hada, M. Ehara, K. Toyota, R. Fukuda, J. Hasegawa, M. Ishida, T. Nakajima, Y. Honda, O. Kitao, H. Nakai, T. Vreven, K. Throssell, J. A. Montgomery, Jr., J. E. Peralta, F. Ogliaro, M. J. Bearpark, J. J. Heyd, E. N. Brothers, K. N. Kudin, V. N. Staroverov, T. A. Keith, R. Kobayashi, J. Normand, K. Raghavachari, A. P. Rendell, J. C. Burant, S. S. Iyengar, J. Tomasi, M. Cossi, J. M. Millam, M. Klene, C. Adamo, R. Cammi, J. W. Ochterski, R. L. Martin, K. Morokuma, O. Farkas, J. B. Foresman and D. J. Fox, Gaussian, Inc., Wallingford CT, 2019.

(3) A. Austin, G. A. Petersson, M. J. Frisch, F. J. Dobek, G. Scalmani and K. Throssell, A Density Functional with Spherical Atom Dispersion Terms. *J. Chem. Theory Comput.* **2012**, *8*, 4989–5007.

(4) Peterson, K. A. Systematically Convergent Basis Sets with Relativistic Pseudopotentials. I. Correlation Consistent Basis Sets for the Post- d Group 13 – 15 Elements. *J. Chem. Phys.* **2003**, *119* (21), *11099*.

(5) Peterson, K. A.; Puzzarini, C. Systematically Convergent Basis Sets for Transition Metals. II. Pseudopotential-Based Correlation Consistent Basis Sets for the Group 11 (Cu, Ag, Au) and 12 (Zn, Cd, Hg) Elements. *Theor. Chem. Acc.* **2005**, *114*, 283–296.

(6) Figgen, D.; Rauhut, G.; Dolg, M.; Stoll, H. Energy-Consistent Pseudopotentials for Group 11 and 12 Atoms: Adjustment to Multi-Configuration Dirac–Hartree–Fock Data. *Chem. Phys.* **2005**, *311*, 227–244.

(7) You, A.; Be, M. A. Y.; In, I. Hartree – Fock-Adjusted Pseudopotentials for Post- d Main Group Elements : Application to PbH and PbO. *J. Chem. Phys.* **2010**, *113* (7), 2563-2569.

(8) Pritchard, B. P.; Altarawy, D.; Didier, B.; Gibson, T. D.; Windus, T. L. New Basis Set Exchange: An Open, Up-to-Date Resource for the Molecular Sciences Community. *J. Chem. Inf. Model.* **2019**, *59*, 4814-4820.

(9) Gorelsky, S. I. AOMix: Program for Molecular Orbital Analysis; Version 6.94, 2018, Http://Www.Sg-Chem.Net/.

(10) Gorelsky, S. I.; Lever, A. B. P. Electronic Structure and Spectra of Ruthenium Diimine Complexes by Density Functional Theory and INDO/S. Comparison of the Two Methods. *J. Organomet. Chem.* **2001**, *635*, 187–196.

(11) NBO 7.0. E. D. Glendening, J. K. Badenhoop, A. E. Reed, J. E. Carpenter, J. A. Bohmann, C. M. Morales, P. Karafiloglou, C. R. Landis and F. Weinhold, Theoretical Chemistry Institute, University of Wisconsin, Madison, WI (2018).

(12) GaussView, Version 6.1, Roy Dennington, Todd A. Keith and John M. Millam, Semichem Inc., Shawnee Mission, KS, 2016.a
